# Apolipoprotein E dysfunction in Alzheimer’s disease: a study on miRNA regulation, glial markers, and amyloid pathology

**DOI:** 10.3389/fnagi.2024.1495615

**Published:** 2024-12-18

**Authors:** Printha Wijesinghe, Hao Ran Li, Zhengyuan Ai, Matthew Campbell, Si Xuan Chen, Jeanne Xi, Wellington Pham, Joanne A. Matsubara

**Affiliations:** ^1^Department of Ophthalmology and Visual Sciences, Faculty of Medicine, Eye Care Centre, The University of British Columbia, Vancouver, BC, Canada; ^2^Department of Radiology and Radiological Sciences, Vanderbilt University Medical Center, Nashville, TN, United States; ^3^Vanderbilt University Institute of Imaging Science, Vanderbilt University Medical Center, Nashville, TN, United States; ^4^Djavad Mowafaghian Centre for Brain Health, The University of British Columbia, Vancouver, BC, Canada

**Keywords:** apolipoprotein E, Alzheimer’s disease, microRNAs, amyloid peptide, astroglia, microglia

## Abstract

**Introduction:**

Apolipoprotein E (ApoE) plays a crucial role in lipid homeostasis, predominantly expressed in astrocytes and to a lesser extent in microglia within the central nervous system (CNS). While the *APOE4* allele is the strongest genetic risk factor for late-onset Alzheimer’s disease (AD), its precise role in AD pathogenesis remains elusive. *Apoe*-knockout (*Apoe*-ko) mice, mice expressing human *APOE4*, and human *APOE4* carriers exhibit similar deficits in lipid metabolism, cognitive and behavioral functions, and neurodegeneration. The retina, as part of the CNS, has been studied to investigate the underlying mechanisms of AD, including neuroinflammation, amyloid aggregation, and neurodegeneration. This study explores ApoE’s role in AD by analyzing brain and eye samples from *Apoe*-ko mice, focusing on identifying potential retinal biomarkers associated with ApoE dysfunction.

**Methods:**

We compared female *Apoe*-ko mice on a regular diet to age-matched C57BL/6J controls at 3 and 9 months. Our investigations included microRNAs (miRNAs), their target messenger RNAs (mRNAs), and selected protein markers, including astroglial (Gfap), microglial/macrophage (Iba1 and Trem2) markers, and amyloid precursor protein (APP)/amyloid-β (Aβ) peptides implicated in AD pathogenesis. We also examined female *Apoe*-ko mice on a high-fat diet versus a regular diet at 9 months for differential miRNA and mRNA expressions.

**Results:**

Our findings demonstrated that miRNA levels were generally lower in 3-month-old *Apoe*-ko mice but increased in 9-month-old mice across five distinct brain regions, as well as in eye tissue and tear fluid. A high-fat diet further enhanced miRNA dysregulation in brain and eye tissues, but not in tear fluid. Target mRNAs were generally higher in the neocortex-hippocampus and eye tissue of 3-month-old *Apoe*-ko mice but decreased with age, except for glial cell mRNAs like *Gfap* and *Aif1*. Protein analysis revealed elevated Gfap expression, and increased APP/Aβ peptide accumulation in the neocortex-hippocampus, including brain endothelial cells at the meninges, as well as in the retina of 9-month-old *Apoe*-ko mice. These findings highlight ApoE’s pivotal role in AD, demonstrating its impact on inflammatory and amyloidogenic/angiogenic miRNA expression, glial homeostasis, and APP/Aβ peptide clearance. The observed upregulation of proinflammatory miR-146a and anti-amyloidogenic/angiogenic miR-15a in 9-month-old *Apoe*-ko mice suggests their potential as tear-based biomarkers for ApoE dysfunction.

## 1 Introduction

In 2020, over 55 million people were living with dementia, a number expected to rise to 78 million by 2030 worldwide. Alzheimer’s disease (AD) is responsible for 60–80% of all dementia cases. The major underlying pathological hallmarks include the accumulation of amyloid-beta (Aβ) species and neurofibrillary tangles composed of paired helical filaments of hyperphosphorylated tau. There are two primary forms: sporadic or late-onset AD, which accounts for over 95% of cases, and familial or early-onset AD, which accounts for the remainder (Bali et al., 2012). Inherited mutations in the amyloid precursor protein (*APP*), presenilin 1 (*PSEN1*) and presenilin (*PSEN2*) genes are linked to familial AD, while the etiology of sporadic AD remains complex, with polymorphisms in apolipoprotein E (*APOE*) and triggering receptor expressed on myeloid cells 2 (*TREM2*) being among the most common genetic risk factors (Guerreiro et al., 2013). Specifically, the *ε4* allele of the *APOE* increases the risk of developing sporadic AD, with individuals carrying one ε4 allele having a 2- to 3-fold increased risk, and those with two *ε4* alleles facing a 10- to 15-fold higher risk (Kim et al., 2009; Wijesinghe et al., 2016; Yamazaki et al., 2019; Narasimhan et al., 2024). ApoE isoforms differentially influence various aspects of AD pathology, including Aβ aggregation and clearance, tau pathology, innate immune response, synaptic integrity, glucose metabolism, cerebrovascular function, and age-related cognitive decline (Kim et al., 2009; Liu et al., 2013; Safieh et al., 2019; Yamazaki et al., 2019; Raulin et al., 2022).

ApoE functions as a ligand for lipoprotein receptors, facilitating lipoprotein clearance (Lo Sasso et al., 2016). In the brain, cholesterol is vital for synapse formation and maintenance, with ApoE playing a critical role in regulating cholesterol homeostasis (Zhang and Liu, 2015). Within the central nervous system (CNS), astrocytes and microglia, which express ApoE, perform essential immune and maintenance functions. However, under disease conditions, these glial cells can become dysfunctional, leading to chronic inflammation and neurodegeneration (Parhizkar and Holtzman, 2022). In mice, the single *Apoe* isoform resembles the human *APOE3* allele (Chen et al., 2012). *Apoe-*knockout (*Apoe*-ko) mice show delayed lipoprotein clearance, resulting in hyperlipoproteinemia, severe hypercholesterolemia, and atherosclerosis (Tamminen et al., 1999; von Holt et al., 2009; De León et al., 2014). Similarly, humans with the *APOE4* allele display elevated levels of total cholesterol, low-density lipoprotein (LDL), and oxidized LDL, which increases their risk of developing atherosclerotic plaques (Wijesinghe et al., 2020; Culleton et al., 2023; Yang et al., 2023). ApoE is also essential for maintaining synaptic integrity, plasticity, and dendritic complexity, as evidenced by studies in *Apoe*-ko mice (Fitz et al., 2015; Lane-Donovan et al., 2016). Both *Apoe*-ko mice and those expressing human *APOE4*, as well as individuals with the *APOE4* allele, exhibit similar impairments in lipid metabolism, cognitive function, and neurodegeneration (Liu et al., 2013; Janssen et al., 2016; Wang et al., 2023; Faraji et al., 2024).

As part of the CNS, the eye’s retina has been studied by us and other researchers to investigate underlying AD mechanisms, including neuroinflammation, amyloid aggregation, and neurodegeneration (Lee et al., 2020; Sidiqi et al., 2020; Xu et al., 2022; Wijesinghe et al., 2023a; Gaire et al., 2024; Hart de Ruyter et al., 2024). Our previous work highlighted the potential of tear-based microRNA (miRNA) biomarkers in AD pathogenesis by analyzing brain, eye, and tear samples from a transgenic AD (APP-PS1) mouse model at two different ages (Wijesinghe et al., 2023b). MiRNAs are small, noncoding, single-stranded RNA molecules, abundant in many mammalian cell types, highly conserved, and believed to target approximately 60% of human genes (Friedman et al., 2009). A single miRNA can target multiple genes, and several miRNAs can target a single gene. MiRNAs are highly stable in the extracellular environment and have emerged as potential biomarkers for diagnostic and prognostic purposes.

Limited information is available regarding miRNA alteration in relation to ApoE dysfunction in AD. A prospective study of blood samples from patients with mild cognitive impairment (MCI) revealed a significant upregulation of miR-146a and miR-181a in individuals who later progressed to AD (Ansari et al., 2019). Furthermore, elevated levels of miR-146a were associated with the presence of the *APOE4* allele, reduced hippocampal volume, and atrophy in the CA1 and subiculum subfields. Cao et al., 2021 established a mechanistic link between *APOE4* genotype-specific alterations in brain miR-195 expression and AD-related phenotypes, including phospholipid dysregulation, cognitive deficits, lysosomal dysfunction, and tau pathology. The authors demonstrated that miR-195 rescued *APOE4*-induced cognitive deficits in *APOE4*+/+ mouse hippocampal tissue and cultured neurons, as well as lysosomal defects in iPSC-derived brain cells from *APOE4*+/+ AD subjects.

*Apoe*-ko mice are recommended for studying ApoE’s role in AD (Piedrahita et al., 1992). Recent studies have shown that these mice exhibit brain network alterations (Stapleton et al., 2023), age-related behavioural changes (Fuentes et al., 2018) and disrupted lipid and protein metabolism (Liu et al., 2024), all associated with late-onset AD. In this study, we analyzed the expression levels of mature microRNAs (miRNAs), their target messenger RNAs (mRNAs), and selected glial proteins along with APP/ Aβ peptides involved in AD pathogenesis in brain and eye samples. We hypothesize that ApoE deficiency alters miRNA and mRNA expression levels, disrupts glial homeostasis and APP/ Aβ peptide clearance.

## 2 Materials and methods

### 2.1 Animals

*Apoe*-ko mice (B6.129P2-*Apoe^tm1Unc^*/J, Strain #002052) and their suggested wildtype controls (C57BL/6 J, Strain # 000664) at two different ages, 3 months and 9 months, were included (*n* = 16, 4 per group, all females). Additionally, *Apoe*-ko mice raised on a high-fat (HFD) diet for 24 weeks (Adjusted Calories Diet - 42% from fat, ENVIGO+++) starting at 4 months of age, and those on a regular diet (RD) were studied at 9 months old (*n* = 4–5 per group, all females). A total of 26 female mice were used in 6 groups: 3-month-old *Apoe*-ko, 9-month-old *Apoe*-ko, 3-month-old control, 9-month-old control, 9-month-old *Apoe*-ko with HFD diet, and 9-month-old *Apoe*-ko with RD, based on the resource equation method (Charan and Kantharia, 2013). *Apoe*-ko mice develop fatty streaks in the proximal aorta at 3 months of age. These lesions increase with age and progress to a more advanced stage, characterized by less lipid but more elongated cells, typical of pre-atherosclerotic lesions. In this study, different brain regions, eye tissues, and tear fluids were used to determine the expression levels of selected miRNAs at 3-month-old and 9-month-old ages. However, target mRNAs and protein markers were determined only in the neocortex-hippocampus and eye tissues.

### 2.2 Sample collection

This was done as previously described (Wijesinghe et al., 2023b). In summary, ketamine hydrochloride (80 mg/kg) and xylazine hydrochloride (10 mg/kg) were administered subcutaneously as anesthesia before tear fluid collection. Tear fluid was collected from both eyes using sterile Schirmer tear test strips and stored at −80°C. Thereafter, the mice were sacrificed, and the brain and eyes were promptly removed. The left hemisphere was dissected into five regions: neocortex with hippocampus (region 1), olfactory bulb (region 2), striatum-thalamus-hypothalamus (region 3), brainstem (region 4), and cerebellum (region 5). The left brain hemisphere and left eye were used for miRNA and total RNA extractions, while the right hemisphere and right eye were used for immunofluorescence staining.

### 2.3 MiRNA-target mRNA interaction

The expression levels of 8 mature miRNAs including miRs -101a-3p, −125b-5p, −140-3p, −146a-5p, −15a-5p, −34a-5p, −342-3p, and -374c-5p were determined (Supplementary Table S1). The mature sequences of these miRNAs are similar between *Mus musculus* and *Homo sapiens*. Our previous work has already tested these miRNAs in the brain and eye samples of transgenic APP-PS1 AD mice (Wijesinghe et al., 2023b).

Target genes of these miRNAs were identified via TargetScan 7.2 (Agarwal et al., 2015) based on conserved seed regions. miRNA- target gene interactions are visualized using Cytoscape version 3.10.1 (Shannon et al., 2003). This includes amyloid beta precursor protein (*App*), presenilin 1 (*Psen1*), beta-site APP cleaving enzyme (*Bace1*), sortilin-related receptor LDLR class A repeats-containing (*Sorl1*), calcium voltage-gated channel subunit alpha1 C (*Cacna1C*), microtubule associated protein tau (*Mapt*), Rho associated coiled-coil containing protein kinase 1 (*Rock1*), glial fibrillary acidic protein (*Gfap*), signal transducer and activator of transcription 3 (*Stat3*), leukemia inhibitory factor (*Lif*), vascular endothelial growth factor A (*Vegfa*), autophagy related 12 (*Atg12*), sirtuin 1 (*Sirt1*), B cell leukemia/lymphoma 2 (*Bcl2*), brain derived neurotrophic factor (*Bdnf*), complement factor h (*Cfh*), and organic cation transporter novel type 1 (*Slc22a4*). Additionally, three target genes associated with Aβ clearance and inflammation, including aquaporin 4 (*Aqp4*), allograft inflammatory factor 1 (*Aif1*), and triggering receptor expressed on myeloid cells 2 (*Trem2*) were included. A total of 20 target mRNAs were screened in the neocortex-hippocampus and eye tissue (Supplementary Table S2).

### 2.4 Functional enrichment pathway analysis

Each miRNA tested in this study underwent functional enrichment pathway analysis, which included identifying target genes, the number of genes involved in each Reactome pathway, and significant *p*-values, based on strong experimental evidence using miRPathDB 2.0 (Kehl et al., 2020). The Database for Annotation, Visualization, and Integrated Discovery (DAVID) (Sherman et al., 2022) was used to determine the functional enrichment of target genes screened in this study, focusing on commonly involved Kyoto Encyclopedia of Genes and Genomes (KEGG) and Reactome pathways. Additionally, target genes of these 8 mature miRNAs, identified by miRTarBase (Huang et al., 2022) and validated through one of the three strong experimental methods (reporter assay, western blot, or qPCR) for *Mus musculus,* were subjected to Reactome pathway analysis.

### 2.5 MiRNA extraction

The protocols were consistent with previous work (Wijesinghe et al., 2023b). miRNAs from tear fluids were extracted individually using the miRNeasy Serum/Plasma Kit, with 200 μL supernatant homogenized in QIAzol lysis reagent containing MS2 RNA. *Cel*-miR-39 RNA oligos were added before chloroform. miRNAs from brain and eye tissues were extracted using the miRNeasy Mini Kit, with eye tissues processed individually and brain tissues pooled by strain, age group and anatomical region. *Cel*-miR-39 was also added to tissue samples before chloroform. miRNA quantity and quality were assessed before cDNA preparation.

### 2.6 Single tube TaqMan advanced miRNA assay

As previously published (Wijesinghe et al., 2023b), the assays involved multiple stages: poly(A) tailing, adaptor ligation, reverse transcription (RT), miR-Amp amplification, and real-time PCR. Each assay began with 2 μL of 10 ng miRNAs extracted from tissue or tear fluid. RT-qPCR was performed using the 7,500 Fast Real-Time PCR System (Applied Biosystems), with each sample and miRNA analyzed in a minimum of three replicates. Normalization was done using the spike-in control *Cel*-miR-39, as described in our previous study (Wijesinghe et al., 2023b).

### 2.7 Individual gene expression assay

Total RNA was extracted from pooled neocortex-hippocampus, and eye tissues (*n* = 4 for *Apoe*-ko and control groups, and n = 4–5 for diet-based groups) using the RNeasy^®^ Mini Kit. cDNA synthesis was performed with the SuperScript™ VILO™ cDNA Synthesis Kit. Approximately 10 ng of cDNA was used for each reaction. RT-qPCR was conducted on a 7,500 Fast Real-Time PCR System. Glyceraldehyde-3-phosphate dehydrogenase (*Gapdh*) was used as a reference gene for data normalization. Three sets of primer pairs were tested for each gene, and the most effective primer pair was selected for the experiment (Supplementary Table S2) (Untergasser et al., 2007). Each target gene was analyzed in at least three replicates per sample.

### 2.8 Screening of 6E10-Gfap and Iba1-Trem2 protein markers in brain sagittal and eye cross sections

The right half of the brain and the right eye globe of the above animals were used for protein expression studies. Six-micrometer-thick mid-sagittal brain and eye cross sections were stained using a double immunofluorescence protocol as described in our previous work (Wijesinghe et al., 2023a). Both *Apoe*-ko mice (*n* = 4 in each age group: 3-month-old and 9-month-old) and control mice (n = 4 in each age group: 3-month-old and 9-month-old) were evaluated. For APP/Aβ peptide detection, an 88% formic acid pretreatment was applied for 5 min. This was followed by antigen retrieval, performed either with 0.05% proteinase K in Tris-EDTA buffer (pH 8.0) for 10 min at room temperature (RT) or by heat-induced antigen retrieval in citrate buffer (pH 6.0) for 10 min at a power level of 800 watts (Wijesinghe et al., 2023a). The screening utilized primary antibodies, including inflammatory markers such as glial fibrillary acidic protein (Gfap, rabbit polyclonal, Cat# Z0334), a marker for astroglia; ionized calcium-binding adapter molecule 1 (Iba1, rabbit polyclonal, Cat# 019–19,741), a marker for microglia/macrophage-specific calcium-binding protein; triggering receptor expressed on myeloid cells 2 (Trem2, rat monoclonal, Cat# MAB17291), a receptor found in microglia/macrophages; and 6E10 (mouse monoclonal, Cat# 803014, 1:200), which reacts to the 1–16 amino acid residues of both Aβ peptides and APP, with an epitope nearly identical in human and mouse species (Wijesinghe et al., 2023a). To validate the 6E10+ APP labeling, we used an additional knockout validated APP antibody (rabbit monoclonal, Cat# A17911) and the 12F4 antibody (mouse monoclonal, Cat# 805501) which is specific to Aβ 1–42 amino acid residues. Secondary antibodies used for fluorescence confocal microscopy were Alexa Fluor^®^ 488 goat anti-rabbit (Cat# 11070), Alexa Fluor™ 647 donkey anti-rabbit (Cat# 711605152), FITC goat anti-rat IgG2b (Cat# A110-111F), Alexa Fluor™ 546 goat anti-mouse IgG1 (Cat# 21123) and Alexa Fluor™ 546 goat anti-rabbit IgG1 (Cat# 11071). Negative control slides were processed without primary antibodies simultaneously.

### 2.9 Fluorescence confocal microscopy and image analysis

Zeiss LSM800 confocal microscope equipped with ZEN 3.7 (blue edition) software was used for image acquisition.

#### 2.9.1 Imaging parameters

Different fluorophores were used for different antibodies: Alexa Fluor^®^ 546 for 6E10, Alexa Fluor^®^ 488 for Gfap, Alexa Fluor^®^ 647 for Iba1, FITC (491 nm) for Trem2, and DAPI (461 nm) for nuclear labelling. Each antibody was imaged at its corresponding wavelength. Confocal settings were kept constant for each marker and negative across the animal groups to maintain consistency and minimize variability.

#### 2.9.2 Magnifications and regions of interest

Brain sagittal and retinal cross sections were imaged at 200x magnification for 6E10-Gfap and Iba1-Trem2 double labelling. Minimum of 4 non-overlapping regions were captured for hippocampus (dentate gyrus (DG), cornu Ammonis 4 (CA4), CA3-CA2 and CA1) and neocortex (prefrontal, frontal, parietal and occipital). Minimum of 2 central, 2 mid and 2 peripheral regions were captured for retina.

#### 2.9.3 Image analysis

ImageJ software was used for the evaluation of immunoreactivity. Two or more independent investigators evaluated the images anonymously, ensuring unbiased analysis.

This protocol ensured thorough and systematic imaging and analysis allowing for reliable assessment of immunoreactivity in brain and retinal sections.

### 2.10 Data analysis

#### 2.10.1 miRNA and mRNA expression analysis

Relative expression levels were determined using the comparative cycle threshold (Ct) method (Schmittgen and Livak, 2008). Ct values were obtained at a constant threshold and baseline settings across the samples and miRNAs or mRNAs. Normalized Ct values were compared across animal groups and time points. The Shapiro–Wilk test was used to assess normal distribution. For miRNA analysis in eye and tear samples, the Kruskal-Wallis test with Dunn’s multiple comparisons test was employed. MiRNA analysis in pooled brain samples, and mRNA analysis in pooled brain and eye samples, were performed using a two-way ANOVA with Bonferroni-corrected multiple comparisons test. The Mann–Whitney test was employed for miRNA analysis in eye and tear samples between high-fat diet and regular diet *Apoe*-ko mice. For miRNA and mRNA analysis in pooled brain samples, an unpaired t-test (2-tailed) was conducted between high-fat diet and regular diet *Apoe*-ko mice. Dysregulated miRNAs were defined by a statistically significant (*p* < 0.05) 2-fold intergroup difference, while differentially expressed target mRNAs were defined by a statistically significant (*p* < 0.05) 1.5-fold intergroup difference.

#### 2.10.2 Protein expression analysis

Pixel data normalized to area was screened for outliers using the ROUT (*Q* = 1%) method across animal groups at two time points. Normality tests were conducted on the cleaned data. Immunoreactivity was analyzed using the Kruskal-Wallis test with Dunn’s multiple comparisons test. The non-parametric Spearman r correlation test was conducted to assess the strength of associations between protein markers.

All statistical analyses and graph generation were performed using GraphPad Prism 10.3.0 (GraphPad Software Inc., San Diego, CA).

## 3 Results

**Figure 1:**
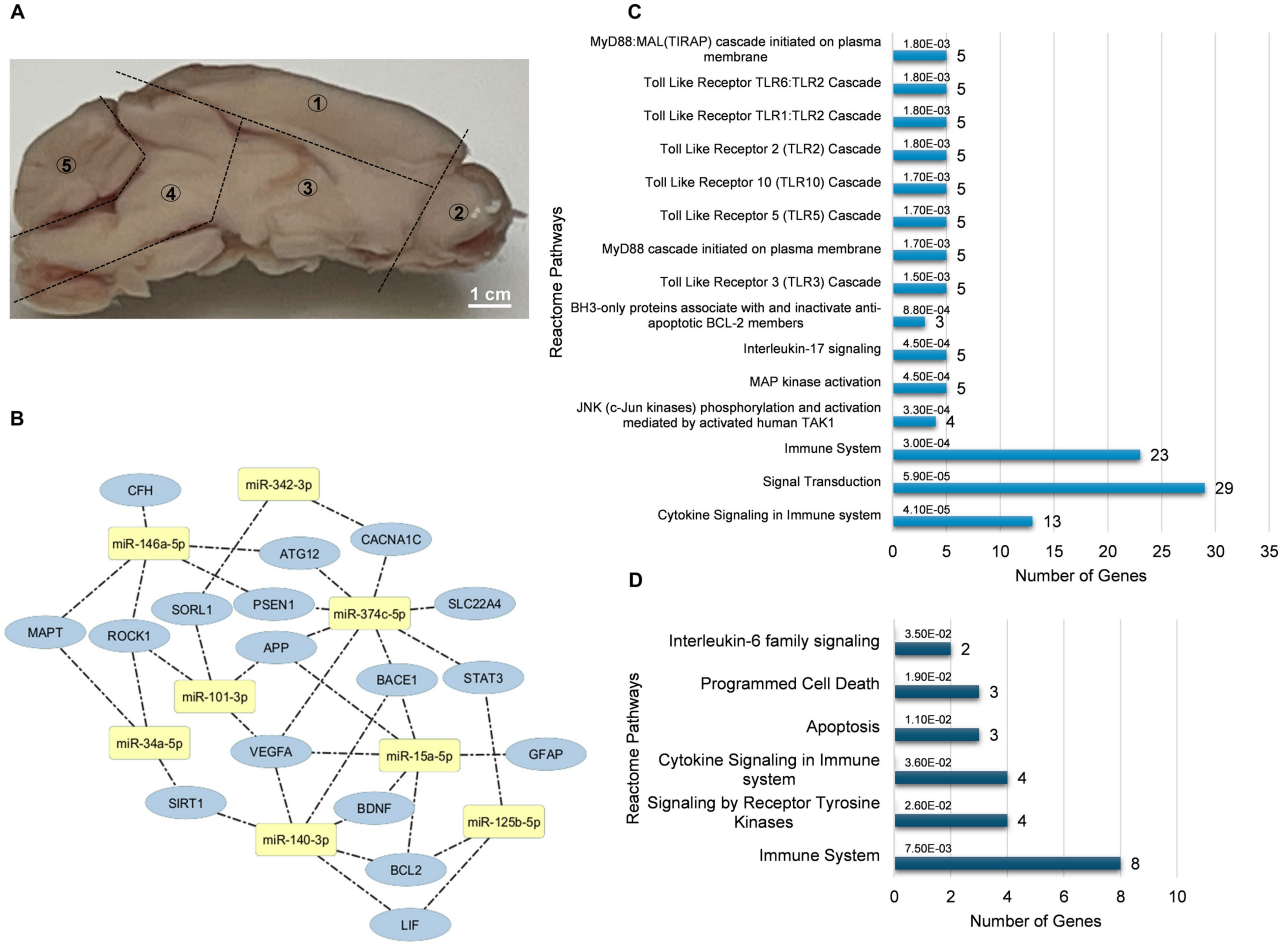
miRNA-mRNA interaction and functional enrichment pathway analysis. **(A)** Representative sagittal section of the mouse brain showing the five distinct regions dissected for miRNA analysis: neocortex-hippocampus (1), olfactory bulb (2), striatum-thalamus-hypothalamus (3), brainstem (4), and cerebellum (5). **(B)** Conserved seed sequence-based interactions between miRNAs and their target mRNAs. **(C)** Reactome pathways enriched for target genes identified by miRTarBase for the miRNAs analyzed in this study. **(D)** Reactome pathways enriched for the target genes tested in this study. Bar graphs display the pathways ranked by the most significant *p* values, along with the number of genes involved.

The left hemisphere of the mouse brain was divided into five distinct regions for miRNA investigation was illustrated in Figure 1A. Conserved seed region-based (TargetScan 7.2) miRNA-target mRNA interactions, which were used to determine expression levels in the neocortex-hippocampus and eye tissue, are illustrated in Figure 1B. Each miRNA’s top three functionally enriched Reatome pathways are summarized in Table 1. These functional enrichments were based on robust experimental evidence; however, they were identified for human miRNAs (e.g., hsa-miR-146a-5p). According to miRPath DB 2.0, none of the mouse miRNAs (e.g., mmu-miR-146a-5p), despite sharing similar sequences, were significantly enriched in any pathway. Using miRTarBase, a total of 74 genes in *Mus musculus* were identified as validated targets of the miRNAs screened in this study. Of these, 58 (78.4%) were enriched in Reactome pathways (Figure 1C). Interestingly, both analyses (for human and mouse miRNAs) revealed significant enrichments for pathways associated with signal transduction, immune system, cytokine signaling, activation of kinases and apoptosis. Reactome pathway analysis for the 20 target genes screened in this study also revealed significant enrichment in pathways related to the immune system, cytokine signaling, activation of kinases and apoptosis (Figure 1D). Additionally, KEGG pathway analysis identified neurodegeneration—multiple diseases (*p* = 0.0011), AD (*p* = 0.004), and the JAK–STAT signaling pathway (*p* = 0.003) as the only significant pathways enriched for the 20 target genes screened in this study.

**Table 1:** Top Reactome pathway enrichments and target genes for tested miRNAs.

MiRNAs	Reactome pathways	Hits	*P* value	Targets
miR-101-3p	Signaling by VEGF	8	0.004	APP,CCND1,CDH5,CDK8,CFTR,CTNNB1,DUSP1,EED, EZH2,FOS,ITGA3,JAK2,MET,MTOR,NLK,NOTCH1, PIK3CB,PRKAB1,PTGER4,RAC1,RAP1B,RHOA,RUNX1, SOX9,SRF,STMN1,VEGFA,VEGFC
	Signal Transduction	28	0.005	
	Signaling by Receptor Tyrosine Kinases	13	0.005	
miR-125b-5p	Erythropoietin activates Phosphoinositide-3-kinase (PI3K)	5	0.009	BBC3, BCL2,BMF,CDKN2A,CDKN2D,E2F2,E2F3,EPO, EPOR,ETS1,HMGA1,HMGA2,JAK2,MAPK14,PIK3CB, PIK3CD,RPS6KA1,STAT3,TP53
	BH3-only proteins associate with and inactivate anti-apoptotic BCL-2 members	4	0.017	
	Cellular Senescence	11	0.017	
miR-140-3p	Integrin cell surface interactions	3	0.002	COL4A1, FN1,GPC1,ITGA6
	Cell surface interactions at the vascular wall	3	0.004	
	Assembly of collagen fibrils and other multimeric structures	2	0.016	
miR-146a-5p	Immune System	35	1.24e-4	CCL5,CCND1,CD40LG,CD80,CFH,CXCL8,DUSP1,EGFR, ERBB4,FADD,ICAM1,IL6,IRAK1,IRAK2,MIF,NFKB1, NOS1,PA2G4,PLAUR,PRKCE,PTGES2,PTGS2,RAC1, RHOA,ROCK1,SIKE1,SLPI,SOS1,SOX2,STAT1,TGFB1, TLR2,TLR4,TRAF6,WASF2
	Innate Immune System	21	0.001	
	Interleukin-10 signaling	6	0.003	
miR-15a-5p	Cell Cycle	10	0.001	AKT3,BRCA1,CCND1,CCND2,CCNE1,CDC25A,CDKN2B, CHEK1,TP53,WEE1
	Cyclin A:Cdk2-associated events at S phase entry	5	0.001	
	Cyclin E associated events during G1/S transition	5	0.001	
miR-34a-5p	Developmental Biology	32	6.31e-4	ATG4A,ATG4B,ATG4C,ATG4D,ATG5,ATG7,AKT1,ANK3,AR,BAX,BECN1,BIRC5,CACNB3,CCND1,CCNE2,CD24,CDK4,CDK6,CDKN2C,CYBB,DLL1,E2F1,E2F3,EPHA5,ERBB2,FOS,FOXP1,GFRA3,HDAC1,HNF4A,HNF4G,IFNB1,IL6R,JAG1,KIT,KLF4,L1CAM,LEF1,MAP2K1,MDM4,MET,MTA2,MYB,MYC,NANOG,NOTCH1,NOTCH2,NR4A2,NUMB,PPARA,POU5F1,RAD51,RICTOR,SIRT1,SMAD4,SOX2,SRC,STX1A,TCF7,TGIF2,TP53,TREM2,WNT1,YY1
	Gene expression (Transcription)	38	6.34e-4	
	Cellular responses to external stimuli	21	0.001	
miR-342-3p	JNK (c-Jun kinases) phosphorylation and activation mediated by activated human TAK1	3	9.46e-4	IKBKG, TAB2,TAB3
	TNFR1-induced NFkappaB signaling pathway	3	9.46e-4	
	activated TAK1 mediates p38 MAPK activation	3	9.46e-4	
miR-374c-5p	None			

The top three Reactome pathways for each miRNA are shown, selected based on strong experimental evidence (including reporter assays, Western blot, and qPCR) and significant P values identified using the miRPathDB 2.0 analysis tool.

To assess the impact of ApoE deficiency, we first investigated miRNAs associated with various processes: proinflammation (−125b, −34a, and -146a), Aβ protein (−101a, −140, −15a, −342, and −374c), tau protein (−101a, −34a, and −146a), apoptosis (−125b, −140, −146a, −15a, and −34a), angiogenesis (−101a, −140, 15a and −374c) and neuroprotection (−140, −146a, −15a, and −374c). The justification for selecting these miRNAs was described in the discussion section.

### 3.1 Dysregulated miRNAs in the neocortex-hippocampus and eye tissue, as well as their circulating levels in tear fluid

**Figure 2:**
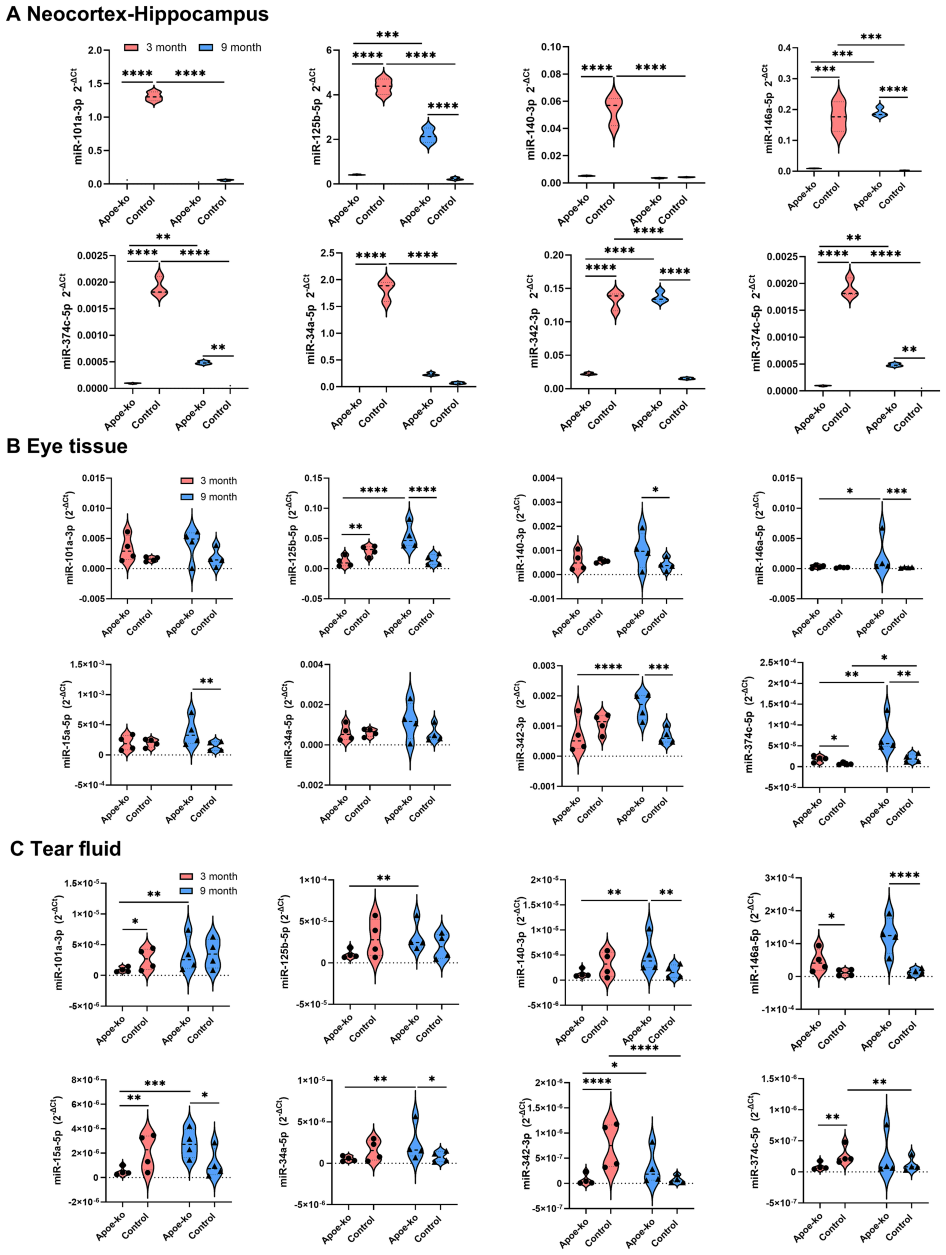
Relative miRNA expression levels in the neocortex-hippocampus, eye tissue and tear fluid. **(A)** Violin plots illustrate 2-^∆Ct^ values in the neocortex-hippocampus of 3-month-old and 9-month-old *Apoe*-ko mice and controls. Pooled neocortex-hippocampus tissue samples (*n* = 4 per group) were used to assess relative miRNA levels. **(B,C)** Violin plots show the distribution of 2^-∆Ct^ values for 3-month-old and 9-month-old *Apoe*-ko mice and controls for eye tissue and tear fluid, respectively. Mean 2^-∆Ct^ values for individual animals (*n* = 4 per group) are overlaid on each plot. Dysregulated miRNAs are defined based on a 2-fold difference between groups and statistical significance at *p < 0.05, ***p < 0.001, and ****p < 0.0001. A two-way ANOVA with Bonferroni-corrected multiple comparisons was used for neocortex-hippocampus analysis, and the Kruskal-Wallis test with Dunn’s multiple comparisons was applied for individual eye and tear fluid analyses.

In this study, we focused primarily on the neocortex-hippocampus, which is the most affected area in AD (Figure 2A; Supplementary Table S3). Eye tissue (Figure 2B; Supplementary Table S4) and tear fluid (Figure 2C; Supplementary Table S5) were also examined to explore the translational potential of retinal biomarkers, with an emphasis on tear-based biomarkers.

In the neocortex-hippocampus of 3-month-old *Apoe*-ko mice, all tested miRNAs were significantly downregulated compared to matched controls (Figure 2A; Supplementary Table S3). In the eye tissue, only two miRNAs showed significant dysregulation (−125b and −374c) between 3-month-old *Apoe*-ko mice and matched controls (Figure 2B and Supplementary Table S4). In the tear fluid, four miRNAs (−101a, −15a, −342, and −374c) were significantly downregulated in 3-month-old *Apoe*-ko mice compared to matched controls (Figure 2C and Supplementary Table S5), with the exception of miR-146a, which showed significant a 4-fold increase (*p* = 0.035). In 9-month-old *Apoe*-ko mice, five miRNAs (−125b, −146a, −15a, −342, and −374c) were significantly upregulated in both the neocortex-hippocampus and eye tissue compared to age-matched controls. Notably, two of these miRNAs (−146a and -15a) were similarly elevated in tear fluid. Additionally, miR-140 in both eye tissue and tear fluid, and miR-34a in tear fluid, were significantly upregulated in 9-month-old *Apoe*-ko mice compared to controls.

Over time, four miRNAs—125b, −146a, −342, and −374c—showed consistent and significant upregulation in the neocortex-hippocampus and eye tissue of *Apoe*-ko mice, with two of them (−125b, and −342) also being reflected in tear fluid. Additionally, miR-15a and miR-374c in neocortex-hippocampus, and miRNAs -101a, −140, −15a, and −34a in tear fluid showed, significant increases. In contrast, all tested miRNAs in the neocortex-hippocampus of control mice were significantly downregulated over time. However, in eye tissues, miR-374c showed significant upregulation, while the other miRNAs did not display any significant changes. Similarly, in tear fluid, miR-342 and miR-374c exhibited significant downregulation, while the other miRNAs remained unchanged.

**Figure 3:**
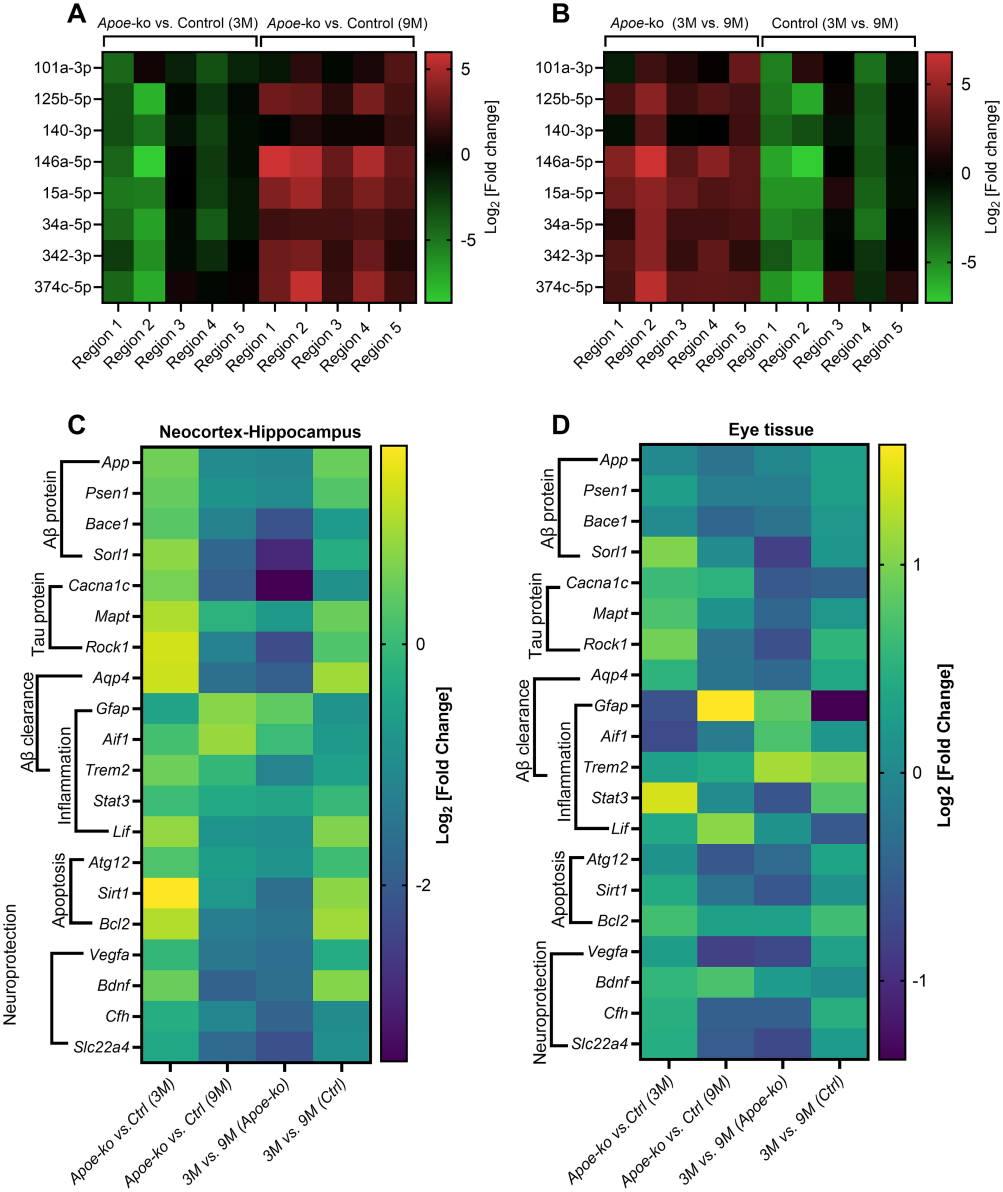
Differential expression of miRNAs and glial mRNAs in 9-month-old *Apoe*-ko mice. **(A,B)** Heatmaps show miRNA levels as log2 [fold change], comparing 3-month-old and 9-month-old *Apoe*-ko mice with controls, as well as age-related changes within the same strains across five different brain regions. **(C,D)** Heatmaps display mRNA levels as log2 [fold change], comparing 3-month-old and 9-month-old *Apoe*-ko mice with controls and tracking changes over time within the same strains for the neocortex-hippocampus and eye tissue, respectively. (Ctrl, control).

Relative miRNA expression levels across different brain regions were compared between *Apoe*-ko mice and controls at 3 and 9 months of age (Figure 3A), as well as within the same group of animals over time (9-month-old vs. 3-month-old, Figure 3B). Overall, miRNA expression levels followed a consistent pattern across the five brain regions, with generally lower levels in 3-month-old *Apoe*-ko mice and higher levels in 9-month-old *Apoe*-ko mice.

After miRNA analysis, we determined target mRNAs associated with Aβ (*App*, *Psen1*, *Bace1*, and *Sorl1*), tau (*Mapt*, *Rock1*, and *Cacna1C*), inflammation (*Gfap*, *Aif1*, *Trem2*, *Lif*, and *Stat3*), Aβ clearance (*Gfap*, *Aqp4*, *Aif1*, and *Trem2*), apoptosis (*Sirt1, Bcl2,* and *Atg12*), and neuroprotection (*Vegfa*, *Bdnf*, *Cfh*, and *Slc22a4*) in the neocortex-hippocampus and eye tissue. Their selection was primarily based on both conserved seed regions (Figure 1C) and existing literature.

### 3.2 Differentially expressed target mRNAs in neocortex-hippocampus and eye tissue

#### 3.2.1 Neocortex-hippocampus

In general, mRNA expression levels were higher in 3-month-old *Apoe*-ko mice compared to matched controls, but lower in 9-month-old *Apoe*-ko mice compared to their respective controls (Table 2; Supplementary Figure S1). Among these, eight target mRNAs (*Sorl1*, *Cacna1c*, *Mapt*, *Rock1*, *Aqp4*, *Lif*, *Sirt1*, and *Bcl2*) were significantly upregulated (above the 1.5-fold cutoff) in 3-month-old *Apoe*-ko mice compared with matched controls. In contrast, fourteen target mRNAs (*App, Psen1, Bace1, Sorl1, Cacna1c, Rock1*, *Aqp4*, *Lif*, *Sirt1*, *Bcl2*, *Vegfa*, *Bdnf*, *Cfh*, and *Slc22a4*) were significantly downregulated (below the 1.5-fold cutoff) in 9-month-old *Apoe*-ko mice compared with 9-month-old controls. Interestingly, the glial cell mRNAs, *Gfap* and *Aif1* were notably upregulated in 9-month-old *Apoe*-ko mice, with significant increases of 1.6-fold (*p* = 0.0002) and 1.8-fold (*p* = 0.0009), respectively. With aging, seventeen out of twenty target mRNAs showed significant downregulation in 9-month-old *Apoe*-ko mice compared to 3-month-old *Apoe*-ko mice, except for *Gfap*, *Aif1*, and *Stat3*.

**Table 2:** Relative mRNA levels in the neocortex-hippocampus of *Apoe*-ko mice and controls.

Target messenger RNAs	Differentially expressed mRNAs									
	(≥1.5-fold intergroup difference and *p* < 0.05)									
	*Apoe*-ko vs. Controls (3-month-old)		*Apoe*-ko vs. Controls (9-month-old)		9-month-old vs. 3-month-old *Apoe*-ko mice		9-month-old vs. 3-month-old controls		*Apoe*-ko HFD vs. *Apoe*-ko RD	
	FC	*P* value	FC	*P* value	FC	*P* value	FC	*P* value	FC	*P* value
*App*	1.46	0.0002	**0.48**	**<0.0001**	**0.46**	**<0.0001**	1.39	0.0007	N/d	
*Psen1*	1.36	0.0001	**0.54**	**<0.0001**	**0.48**	**<0.0001**	1.21	0.0045	N/d	
*Bace1*	1.24	0.0368	**0.44**	**0.0036**	**0.22**	**<0.0001**	**0.62**	**0.0022**	**11.13**	**<0.0001**
*Sorl1*	**1.71**	**<0.0001**	**0.28**	**<0.0001**	**0.13**	**<0.0001**	0.80	0.0228	0.84	0.016
*Cacna1c*	**1.52**	**0.0003**	**0.26**	**0.0019**	**0.09**	**<0.0001**	**0.53**	**0.0006**	N/d	
*Mapt*	**2.04**	**<0.0001**	0.88	0.2137	**0.60**	**<0.0001**	1.40	0.0025	**2.57**	**0.0002**
*Rock1*	**2.44**	**<0.0001**	**0.43**	**0.0013**	**0.20**	**<0.0001**	1.17	0.8788	**0.16**	**<0.0001**
*Aqp4*	**2.38**	**<0.0001**	**0.33**	**<0.0001**	**0.26**	**<0.0001**	**1.89**	**<0.0001**	N/d	
*Gfap*	0.70	0.001	**1.65**	**0.0002**	1.31	0.0072	**0.56**	**<0.0001**	**30.71**	**0.0027**
*Aif1*	1.09	>0.9999	**1.82**	**0.0009**	1.01	>0.9999	**0.61**	**0.0038**	1.21	0.4631
*Trem2*	1.42	0.0271	0.95	>0.9999	**0.44**	**0.0005**	0.67	0.0899	**3.97**	**0.0032**
*Stat3*	1.02	>0.9999	0.77	0.2951	0.73	0.1291	0.97	>0.9999	**0.04**	**<0.0001**
*Lif*	**1.75**	**0.0257**	**0.56**	**0.0376**	**0.51**	**0.0124**	1.60	0.0825	**0.11**	**0.0004**
*Atg12*	1.17	>0.9999	0.64	0.1393	**0.57**	**0.03**	1.03	>0.9999	**13.80**	**<0.0001**
*Sirt1*	**3.12**	**<0.0001**	**0.59**	**0.0429**	**0.32**	**<0.0001**	**1.70**	**0.0397**	**27.77**	**<0.0001**
*Bcl2*	**2.09**	**<0.0001**	**0.40**	**<0.0001**	**0.36**	**<0.0001**	**1.88**	**0.0001**	N/d	
*Vegfa*	0.95	0.3774	**0.37**	**<0.0001**	**0.32**	**<0.0001**	0.80	0.0003	**12.62**	**0.0166**
*Bdnf*	1.38	0.0011	**0.27**	**<0.0001**	**0.32**	**<0.0001**	**1.64**	**<0.0001**	**0.15**	**0.0002**
*Cfh*	0.81	0.0047	**0.46**	**0.0004**	**0.28**	**<0.0001**	**0.50**	**<0.0001**	**13.49**	**<0.0001**
*Slc22a4*	0.74	0.0431	**0.30**	**0.0056**	**0.21**	**0.0002**	**0.52**	**0.0009**	**27.51**	**0.0002**

*Apoe-ko* mice (*n* = 4 per group, females) and controls (C57BL/6 J) were compared at 3-month-old and 9-month-old ages, as well as within strains over time (2-way ANOVA with Bonferroni corrected multiple comparison test). Additionally, *Apoe-ko* mice on a high-fat diet (HFD) versus a regular diet (RD) (*n* = 4–5 per group, females) were compared at 9 months (2-tailed unpaired *t* test). Differentially expressed mRNAs are highlighted in bold based on *P* < 0.05 and an intergroup difference greater than 1.5-fold (FC, Fold change; N/d, Not determined).

#### 3.2.2 Eye tissue

In the 3-month-old group, four target mRNAs (*Sorl1, Cacna1c, Rock1,* and *Bcl2*) showed significant upregulation in *Apoe*-ko mice compared to controls (Table 3 and Supplementary Figure S2). Importantly, the glial cell mRNAs, *Gfap* and *Aif1* were notably downregulated in 3-month-old Apoe-ko mice, with significant decreases of 1.6-fold (*P* = 0.0022) and 1.7-fold (*P* = 0.0396), respectively. In the 9-month-old group, *Bdnf* was significantly upregulated, while *Vegfa* significantly downregulated in *Apoe*-ko mice compared to matched controls. With aging, *Gfap* and *Aif1* were significantly upregulated, while *Sorl1*, *Sta3*, *Vegfa* and *Slc22a4* were was significantly downregulated in *Apoe*-ko mice. Interestingly, *Gfap* was significantly downregulated in 9-month-old controls compared to 3-month-old controls.

**Table 3:** Relative mRNA level in the eye tissues of *Apoe*-ko mice and controls at 3-month-old and 9-month-old ages and based on diet.

Target messenger RNAs	Differentially expressed mRNAs									
	(≥1.5-fold intergroup difference and p < 0.05)									
	*Apoe*-ko vs. Controls (3-month-old)		*Apoe*-ko vs. Controls (9-month-old)		9-month-old vs. 3-month-old *Apoe*-ko mice		9-month-old vs. 3-month-old controls		*Apoe*-ko HFD vs. *Apoe*-ko RD	
	FC	*P* value	FC	*P* value	FC	*P* value	FC	*P* value	FC	*P* value
*App*	1.00	>0.9999	0.83	<0.0001	0.99	>0.9999	1.20	<0.0001	0.76	0.0001
*Psen1*	1.20	0.0025	0.90	0.0549	0.91	0.0807	1.21	0.0019	0.87	0.0069
*Bace1*	1.02	>0.9999	0.74	0.0129	0.83	0.1866	1.14	0.3821	**0.58**	**0.0003**
*Sorl1*	**2.02**	**0.0013**	1.02	>0.9999	**0.57**	**0.0037**	1.12	>0.9999	0.81	0.0233
*Cacna1c*	**1.54**	**0.0076**	1.43	0.0063	0.67	0.0067	0.72	0.0072	1.10	0.5065
*Mapt*	1.67	0.3688	1.09	>0.9999	0.75	0.4591	1.14	>0.9999	0.79	0.0257
*Rock1*	**1.93**	**0.0094**	0.83	>0.9999	0.63	0.1093	1.46	>0.9999	0.88	0.2373
*Aqp4*	1.44	<0.0001	0.84	0.2547	0.76	0.0006	1.30	0.0107	0.89	0.0936
*Gfap*	**0.64**	**0.0022**	2.98	0.1545	**1.79**	**0.0107**	**0.38**	**0.0231**	**0.50**	**0.0015**
*Aif1*	**0.60**	**0.0396**	0.89	0.0004	**1.67**	**0.0055**	1.13	0.0015	0.78	0.0013
*Trem2*	1.22	0.1281	1.34	>0.9999	2.26	0.1192	2.05	>0.9999	0.98	0.5532
*Stat3*	2.63	>0.9999	1.02	0.4955	**0.66**	**0.0143**	1.71	0.1014	0.82	0.1007
*Lif*	1.30	0.0002	2.09	>0.9999	1.08	0.0126	0.67	0.0421	1.46	0.0003
*Atg12*	1.08	0.343	0.67	0.0041	0.78	>0.9999	1.26	0.2646	0.76	0.0022
*Sirt1*	1.33	0.1635	0.83	<0.0001	0.67	0.0002	1.07	<0.0001	0.67	0.0159
*Bcl2*	**1.60**	**0.0005**	1.23	0.0226	1.22	<0.0001	1.59	>0.9999	0.93	0.394
*Vegfa*	1.18	0.0003	**0.57**	**0.0084**	**0.60**	**0.0108**	1.25	0.0003	0.72	0.0072
*Bdnf*	1.47	0.0623	**1.65**	**<0.0001**	1.17	0.0002	1.04	0.0129	**1.51**	**0.0006**
*Cfh*	1.39	0.0033	0.71	0.0003	0.71	0.1211	1.40	>0.9999	**0.54**	**0.001**
*Slc22a4*	1.36	0.2848	0.70	0.3065	**0.60**	**0.0473**	1.17	>0.9999	1.07	0.5975

*Apoe-ko* mice (*n* = 4 per group, females) and controls (C57BL/6 J) were compared at 3-month-old and 9-month-old ages, as well as within strains over time (2-way ANOVA with Bonferroni corrected multiple comparison test). Additionally, *Apoe-ko* mice on a high-fat diet (HFD) versus a regular diet (RD) (*n* = 4–5 per group, females) were compared at 9 months (2-tailed unpaired t test). Differentially expressed mRNAs are highlighted in bold based on *P* < 0.05 and an intergroup difference greater than 1.5-fold. (FC, Fold change).

Heatmaps generated for genes expressed in both the neocortex-hippocampus (Figure 3C) and eye tissue (Figure 3D) indicated differential expression, particularly of glial cell mRNAs such as *Gfap* and *Aif1* in 9-month-old *Apoe*-ko mice, with their regulation direction being opposite to that of other genes.

### 3.3 Impact of a high-fat diet on the expression levels of miRNAs and mRNAs

High-fat diet and ApoE deficiency have been studied in relation to retinal degenerative diseases. Because the retina is considered a surrogate tissue for studying AD, we investigated miRNA and mRNA levels in both the neocortex-hippocampus and eye tissue, as well as circulating miRNAs in tear samples, based on diet.

**Figure 4:**
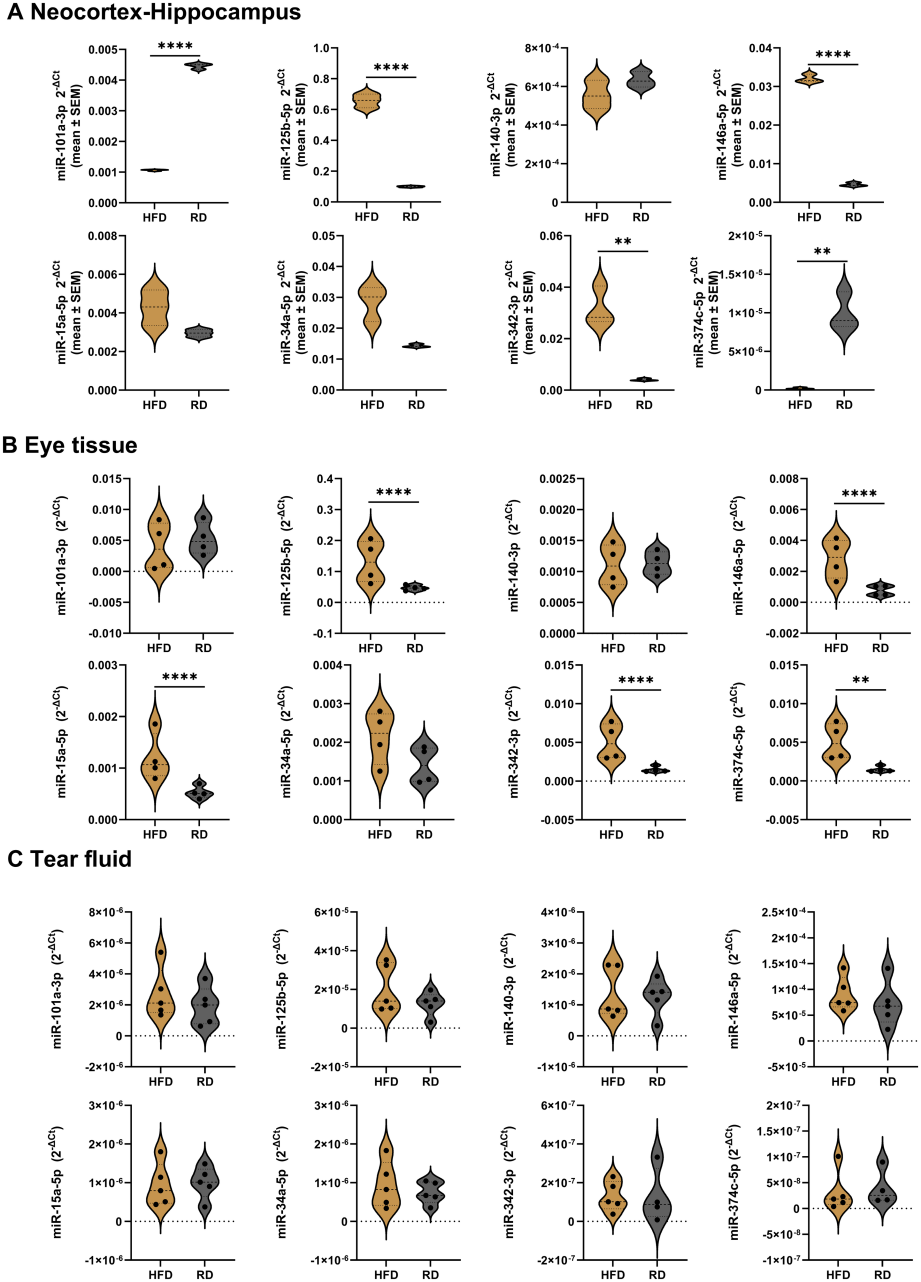
Relative miRNA expression levels in the neocortex-hippocampus, eye tissue and tear fluid of high fat diet *Apoe*-ko mice compared with regular diet *Apoe*-ko mice. **(A)** Violin plots illustrate the distribution of 2^-∆Ct^ values for high-fat and regular diet 9-month-old *Apoe*-ko mice in pooled neocortex-hippocampus tissue samples (*n* = 4–5 per group). Violin plots also show the distribution of 2^-∆Ct^ values between high-fat and regular diet 9-month-old *Apoe*-ko mice for **(B)** eye tissue and **(C)** tear fluid. The mean 2^-∆Ct^ values for individual animals (*n* = 4–5 per group) are overlaid on each plot. Dysregulated miRNAs are defined based on a 2-fold or greater difference between groups and statistical significance at **p < 0.01, and ****p < 0.0001 (2-tailed unpaired *t*-test for pooled neocortex-hippocampus, and the Mann–Whitney test for eye and tear samples). (HFD, high fat diet; RD, regular diet).

Our findings in mice on a high-fat diet indicated that in the neocortex-hippocampus (Figure 4A; Supplementary Table S3) and eye tissue (Figure 4B; Supplementary Table S4), the inflammatory miRNAs -125b, −146a, and −342 were significantly upregulated, while the anti-amyloidogenic/angiogenic miRNAs -101a, −15a, and −374c were significantly dysregulated, showing downregulation in the neocortex-hippocampus (−101a and −374c) and upregulation (−15a and −374c) in the eye tissues. However, none of the tested miRNAs showed significant differences in the tears (Figure 4C; Supplementary Table S5).

The majority of the target mRNAs tested showed no significant differences in the eye tissue of mice on a high-fat diet compared to those on a regular diet. However, *Bace1*, *Gfap*, and *Cfh* were significantly downregulated, while *Bdnf* was significantly upregulated in the high-fat diet mice (Table 3; Supplementary Figure S3). We were unable to determine all 20 target genes in the neocortex-hippocampus. Of the 15 mRNAs assessed, *Bace1*, *Mapt*, *Gfap*, *Trem2*, *Atg12*, *Sirt1*, *Vegfa*, *Cfh*, and *Slc22a4* were significantly upregulated, while *Rock1*, *Stat3*, *Lif*, and *Bdnf* were significantly downregulated (Table 2).

Based on the differential expressions of target mRNAs, glial cells were notably altered in the *Apoe*-ko mice with ageing in both neocortex-hippocampus and eye tissue (Figures 3C,D). In addition to impaired lipid metabolism, the accumulation of APP/Aβ peptides could contribute to aberrant glial cell expression. Therefore, we examined glial cell proteins Gfap, Iba1, and Trem2, along with 6E10+ APP/Aβ peptides, in the neocortex-hippocampus and eye tissue.

### 3.4 ApoE deficiency disrupts glial homeostasis, leading to APP/Aβ peptide accumulation in neocortex-hippocampus

**Figure 5:**
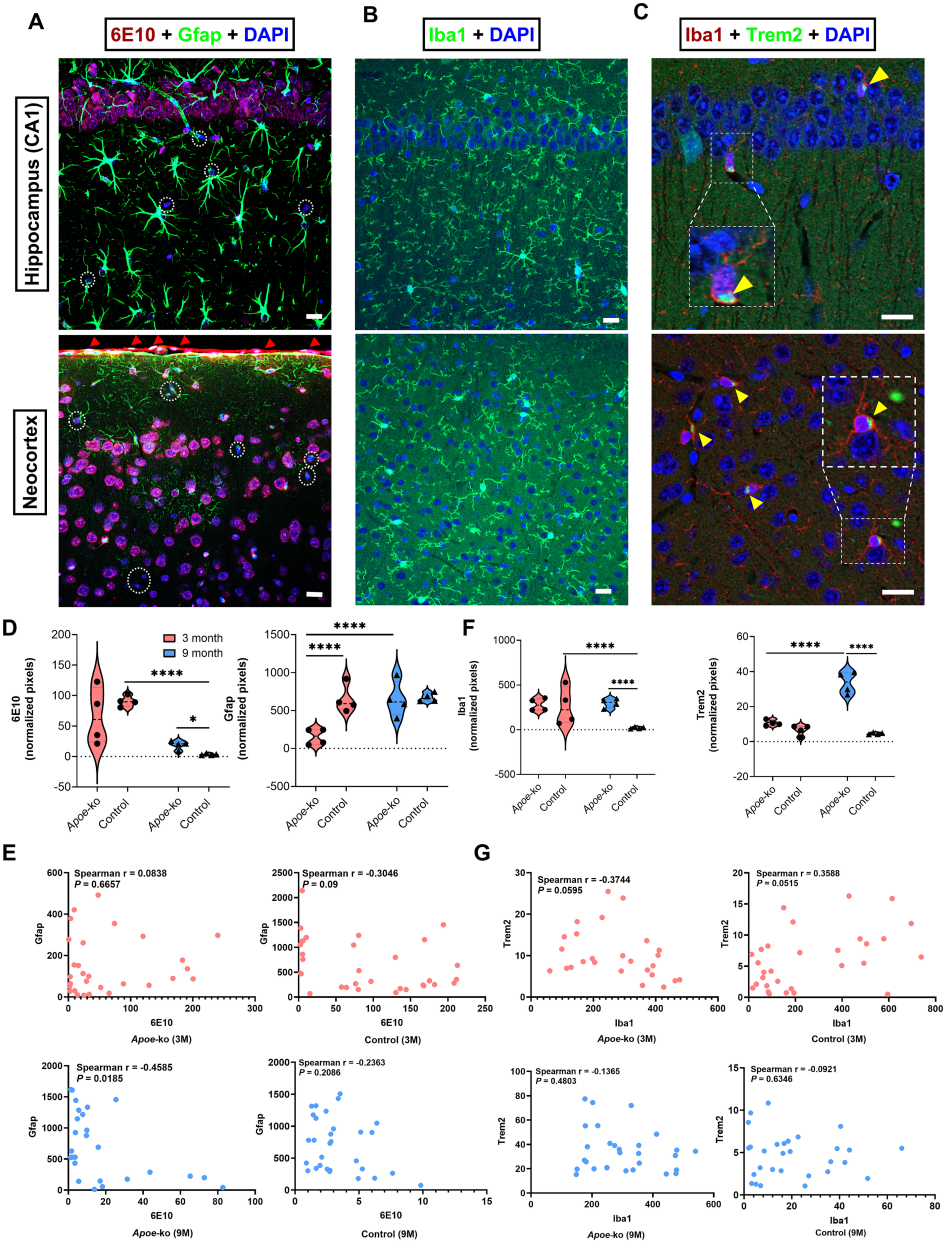
Changes in glial cell expression in relation to APP/Aβ peptide levels in the neocortex-hippocampus. **(A)** Intraneuronal accumulation of 6E10+ APP/Aβ peptides and Gfap + astroglia in the hippocampal CA1 region and neocortex. 6E10+ labelling was also detected in brain endothelial cells at the meninges (indicated by red arrowheads). White dashed circles indicate 6E10-negative cells. **(B,C)** show Iba1+ microglia, and the colocalization of Iba1+ microglia with Trem2+ receptors (zoomed-in, white dashed boxes and yellow arrowheads) in the CA1 region of the hippocampus and neocortex. **(D,F)** illustrate comparisons of 6E10-Gfap and Iba1-Trem2 immunoreactivities between *Apoe*-ko mice and controls at both 3 and 9 months of age, as well as comparisons within the same strains over time. Violin plots display the distribution of normalized pixel values (with outliers removed), and individual animal scores (*n* = 4 per group, all females) are overlaid. Significant differences are indicated by *p* values: *p < 0.05 and ****p < 0.0001, determined using the Kruskal-Wallis test with Dunn’s multiple comparisons test. **(E,G)** show correlations (Spearman’s r) between 6E10-Gfap and Iba1-Trem2 for *Apoe*-ko mice and controls, with *p* values provided for both 3- and 9-month age groups. (Scale bar: 20 μm).

Intraneuronal accumulations of 6E10+ APP/Aβ peptides were identified in the neocortex-hippocampus, with immunoreactivity varying across groups (Figure 5A; Supplementary Table S6). Brain endothelial cells at the meninges also stained positively for APP/Aβ peptides (red arrowheads, Figure 5A). 3-month-old *Apoe*-ko mice showed no significant difference compared to controls, but 9-month-old *Apoe*-ko mice had significantly higher levels (*p* = 0.023) (Figure 5D). Gfap immunoreactivity was significantly lower in 3-month-old *Apoe*-ko mice (*p* < 0.0001) than in controls, while no difference was seen in 9-month-old mice (Figure 5D). No age-related change in 6E10 immunoreactivity was observed in *Apoe*-ko mice, though controls showed a significant reduction (*p* < 0.0001). Conversely, Gfap immunoreactivity significantly changed with aging in *Apoe*-ko mice (*p* < 0.0001), with no such difference in controls. Spearman’s rank correlation revealed a significant negative association between 6E10 and Gfap immunoreactivities in 9-month-old *Apoe*-ko mice (*p* = 0.0185, *r* = −0.4585), but not in 3-month-old mice or controls (Figure 5E).

Supplementary Figures S4A–K illustrated differences in APP/Aβ peptide and astroglia expression in the neocortex-hippocampus of *Apoe*-ko and control mice at 3 and 9 months of age. Higher-magnification images revealed 6E10+ APP/Aβ peptides within neurons (Supplementary Figures S4B,F, indicated by white dashed box and white arrowheads) and endothelial cells at the meninges (Supplementary Figure S4H, indicated by red arrowheads), while white dashed circles marked 6E10-negative cells. The presence of 6E10+ APP in neurons (Supplementary Figures S4C,G, indicated by white dashed box and white arrowheads) and endothelial cells (Supplementary Figure S4I, indicated by red arrowheads) was confirmed using a rabbit monoclonal APP antibody. Additionally, the 12F4 antibody showed positive signals in brain endothelial cells at the meninges, but not within neurons (Supplementary Figures S4D,J, indicated by red arrowheads).

Iba1 expression and its colocalization with the Trem2 were observed in the neocortex-hippocampus (Figures 5B,C). Iba1+ microglia expressing Trem2 receptors were indicated using zoomed white dashed boxes and yellow arrowheads (Figure 5C). Both Iba1 and Trem2 immunoreactivities were significantly higher in 9-month-old *Apoe*-ko mice (*p* < 0.0001), with no significant difference in 3-month-old mice compared to age-matched controls (Figure 5F and Supplementary Table S6). Iba1 levels remained stable with aging in *Apoe*-ko mice but decreased significantly in controls (*p* < 0.0001). Conversely, Trem2 immunoreactivity increased significantly in *Apoe*-ko mice (*p* < 0.0001), remaining unchanged in controls. Spearman’s correlation showed a significant positive association between Iba1 and Trem2 in 3-month-old controls (*p* = 0.05, *r* = 0.3588), but not in *Apoe*-ko mice or 9-month-old controls (Figure 5G).

Supplementary Figures S5A–E was provided to show Trem2 expression with and without microglial colocalization in the neocortex-hippocampus of *Apoe*-ko and control mice at 3 and 9 months of age. Enlarged merged images (Supplementary Figures S5A–C) demonstrated the colocalization of microglia expressing Trem2 receptors (indicated by yellow arrowheads) and cells expressing only Trem2 (indicated by white dashed circles) in the dentate gyrus, CA1 region, and neocortex, respectively.

Overall, 6E10+ APP/Aβ peptides and the microglia/macrophage proteins Iba1 and Trem2 were significantly increased in the neocortex-hippocampus of 9-month-old *Apoe*-ko mice compared to 9-month-old controls. Within strains, Gfap and Trem2 levels showed an age-related increase in *Apoe*-ko mice, while 6E10+ APP/Aβ peptides and Iba1 levels exhibited a significant age-related reduction in controls.

### 3.5 ApoE deficiency disrupts glial homeostasis, leading to APP/Aβ peptide accumulation in eye tissue

**Figure 6:**
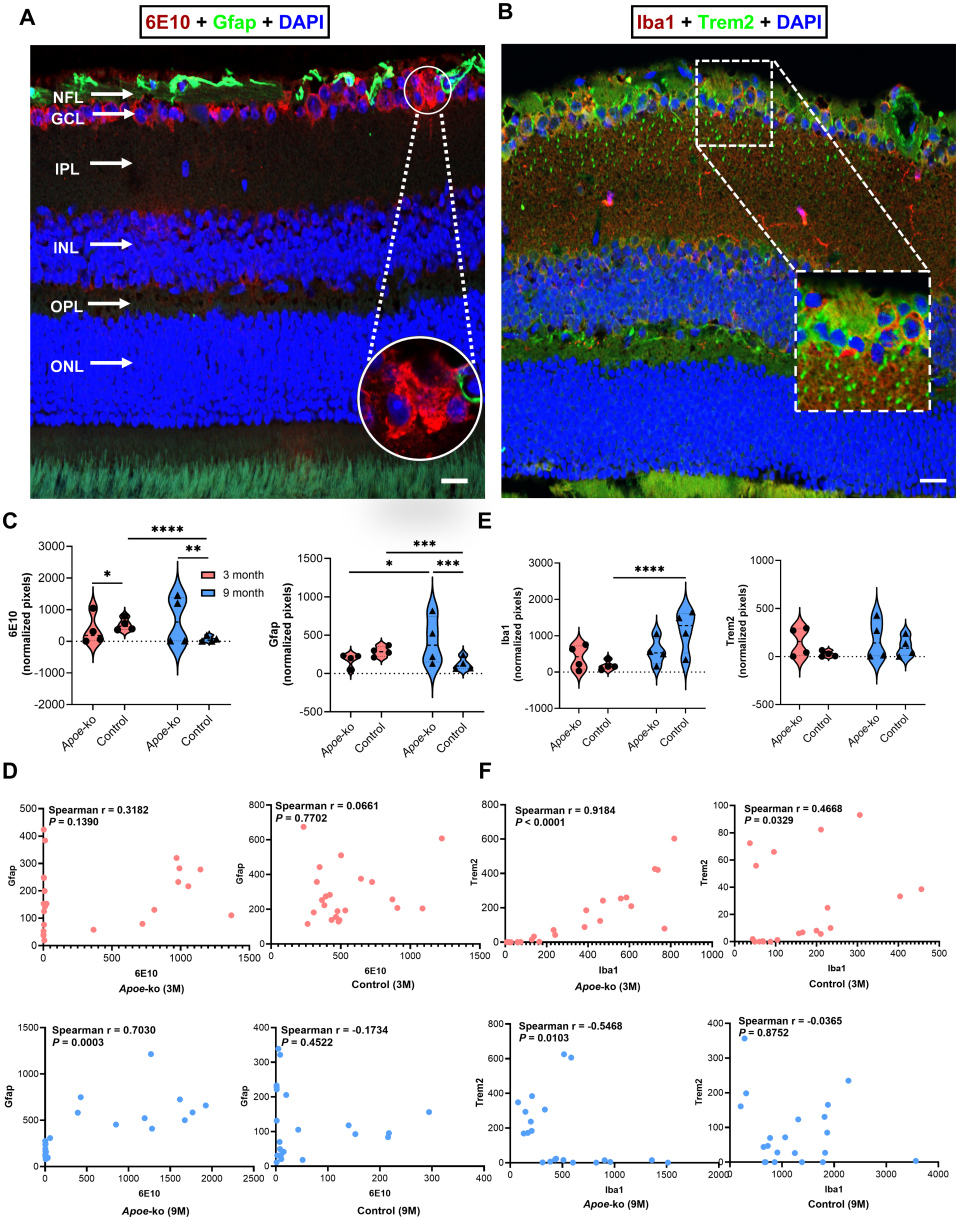
Changes in glial cell expression in relation to APP/Aβ peptide levels in the retina. **(A)** Illustration of 6E10+ APP/Aβ peptides and Gfap + astroglia, focusing on the GCl, with a zoomed-in white circle indicating 6E10+ Aβ plaque-like deposition 6E10+ Aβ. **(B)** Illustration of Iba1+ microglia with and without Trem2+ receptors in the retina, focusing on the NFL-GCL; the zoomed-in white dashed box indicates colocalization. **(C,E)** Comparisons of 6E10-Gfap and Iba1-Trem2 immunoreactivity between *Apoe*-ko mice and controls at both 3 and 9 months of age, as well as comparisons within the same strains over time. Violin plots display the distribution of normalized pixel values (outliers removed), with individual animal scores (n = 4 per group, all females) overlaid. Significant differences are indicated by *p* values: *p < 0.05, **p < 0.01, ***p < 0.001 and ****p < 0.0001, determined using the Kruskal-Wallis test with Dunn’s multiple comparisons test. **(D,F)** Correlations (Spearman’s r) between 6E10-Gfap and Iba1-Trem2 for *Apoe*-ko mice and controls, with *p* values provided for both 3-month-old and 9-month-old age groups. (Scale bar: 20 μm) (NFL, nerve fiber layer; GCL, ganglion cell layer; IPL, inner plexiform layer; INL, inner nuclear layer; OPL, outer plexiform layer; ONL, outer nuclear layer).

Our immunolabeling data confirmed intraneuronal accumulation of 6E10+ APP/Aβ peptides within the retinal layers (Figure 6A). Notably, 9-month-old *Apoe*-ko mice displayed 6E10+ Aβ plaque-like deposition in the inner retina (Figure 6A, zoomed-in white circle). However, further validation with a larger number of samples and different antibodies for Aβ plaques is necessary. 3-month-old *Apoe*-ko mice exhibited significantly lower 6E10 immunoreactivity than controls (*p* = 0.018) (Figure 6C; Supplementary Table S7). In 9-month-old *Apoe*-ko mice, both 6E10 (*p* = 0.009) and Gfap (*p* = 0.0001) immunoreactivities were significantly higher compared to controls. Over time, 6E10 levels remained the same, but Gfap increased significantly in 9-month-old *Apoe*-ko mice (*p* = 0.026). In contrast, 6E10 and Gfap immunoreactivities were significantly reduced in 9-month-old controls (*p* < 0.0001 and *p* = 0.0008). Spearman’s correlation revealed a moderate positive association between 6E10 and Gfap immunoreactivities in 9-month-old *Apoe*-ko mice (*p* = 0.0003, *r* = 0.7030), with no significant correlations in 3-month-old *Apoe*-ko mice or controls (Figure 6D).

Supplementary Figures S6A–F was provided to show the differences in 6E10+ APP/Aβ peptide and astroglial expression in the retina of *Apoe*-ko and control mice at 3 and 9 months of age. Higher magnification images illustrated 6E10+ APP/Aβ peptide accumulation predominantly within the GCL (Supplementary Figure S6B). The presence of APP, predominantly within the GCL, was validated using a rabbit monoclonal APP antibody (Supplementary Figure S6C). The white dashed circles indicated a diffuse plaque-like depositions within the GCL in panels 6B and C. Additionally, 12F4 showed positive signals at the ILM but not within the neurons (Supplementary Figure S6D). The white dashed circle indicated a 12F4+ extracellular Aβ plaque-like deposition within the INL in panel D.

Iba1 and Trem2 colocalization were primarily observed in the NFL-GCL (Figure 6B, zoomed-in white dashed box). Iba1 and Trem2 immunoreactivities showed no significant differences in 3-month-old or 9-month-old *Apoe*-ko mice compared to age-matched controls (Figure 6E and Supplementary Table S7). Within A*poe*-ko mice, both Iba1 and Trem2 levels remained stable over time (Figure 6E and Supplementary Table S7). In controls, Iba1 levels increased significantly (*p* < 0.0001), while Trem2 levels remained unchanged. Spearman’s correlation revealed positive associations between Iba1 and Trem2 immunoreactivities in 3-month-old *Apoe*-ko mice (*p* < 0.0001, *r* = 0.9184) and 3-month-old controls (*p* = 0.033, *r* = 0.4668). However, in 9-month-old *Apoe*-ko mice, a significant negative association was observed (*p* = 0.0103, *r* = −0.5468), with no significant correlation in 9-month-old controls (Figure 6F).

Supplementary Figures S7A,B illustrated Trem2 expression with and without microglia/macrophage colocalization within the retinal layers of *Apoe*-ko and control mice at 3 and 9 months of age. Trem2 and Iba1+ microglia/macrophage expression, with or without colocalization, was predominantly identified in the NFL-GCL.

Overall, 6E10+ APP/Aβ peptides and Gfap levels increased significantly in the retina of 9-month-old *Apoe*-ko mice compared to age-matched controls. In contrast, 6E10+ APP/Aβ peptides and Gfap levels were significantly reduced, while Iba1 expression was significantly increased, in 9-month-old controls compared to 3-month-old controls.

## 4 Discussion

Effective therapies for AD remain elusive due to its complex, multifactorial nature. Despite the strong risk associated with the *APOE4* allele, the precise role of ApoE in AD remains unclear. Previous studies have demonstrated that ApoE, a key mediator of lipid transport in the brain, is downregulated in astrocytes in AD, indicating an imbalance in lipid metabolism (Grubman et al., 2019; Mathys et al., 2019). ApoE has anti-inflammatory and Aβ-metabolizing effects, making it a promising therapeutic target for AD (Komai et al., 2024). To our knowledge, this is the first study to comprehensively investigate molecular and pathological changes in both brain and eye samples of *Apoe*-ko mouse model.

In our study, miRNA expression levels exhibited a consistent pattern across five distinct brain regions, with generally lower levels in 3-month-old *Apoe*-ko mice and higher levels in 9-month-old *Apoe*-ko mice. In contrast, target mRNAs were generally higher in 3-month-old *Apoe*-ko mice but decreased with age, except for glial cell mRNAs such as *Gfap* and *Aif1*, which did not follow this trend. Protein analysis revealed significantly upregulated 6E10+ APP/Aβ peptides in both the neocortex-hippocampus and retina of 9-month-old *Apoe*-ko mice compared to matched controls. Additionally, with aging, GFAP levels increased significantly in both the neocortex-hippocampus and the retina of *Apoe*-ko mice, while 6E10+ APP/Aβ peptides were significantly reduced in both the neocortex and hippocampus of control mice. These findings demonstrate the impact of ApoE dysfunction through the dysregulation of inflammatory and amyloidogenic/angiogenic miRNAs, the differential expression of glial cell mRNAs and proteins, and the increased accumulation of APP/Aβ peptides.

Our selection of miRNAs was guided by relevant literature and TargetScan analyses. miR-101-3p, which shares conserved seed regions with *APP*, *SORL1*, and *VEGFA*, is a well-known anti-amyloidogenic and angiogenic miRNA abundantly present in the brain (Rogaeva et al., 2007; Shao et al., 2010; Vilardo et al., 2010; Long and Lahiri, 2011; DeRosa et al., 2022; Fu et al., 2024). Similarly, miR-15a-5p and its family members (miRs −16 and −195), which share conserved seed regions with *APP*, *BACE1*, and *VEGFA*, have demonstrated anti-amyloidogenic and angiogenic properties (Liu et al., 2019; Zhang et al., 2020; Cao et al., 2021; Sun et al., 2021; Murgia et al., 2022). The proinflammatory miRNAs −125b-5p, −146a-5p, and −34a-5p are implicated in AD and retinal degeneration through immune-mediated pathogenic responses (Bhattacharjee et al., 2016; Zhao et al., 2016; Pogue and Lukiw, 2018; Fan et al., 2020). miR-342-3p, which shares conserved seed regions with *SORL1* and *CACNA1C*, has shown consistent upregulation in APP-PS1 mouse brain tissue over time (Wang et al., 2017) as well as in hippocampal samples from human AD patients and 3xTg AD mice (Fu et al., 2019). miR-342 is also known for its anti-inflammatory properties (Zheng et al., 2023; Zhao and Li, 2024). We also selected miRNAs −140-3p and -374c-5p based on our previous study (Wijesinghe et al., 2023b). miR-140-3p, which is abundant in the brain (Shao et al., 2010), shares conserved seed regions with *BACE1*, *BCL2*, and *SIRT1*. miR-374c-5p targets several AD-associated genes, including *APP*, *BACE1*, *PSEN1*, *CACNA1C*, *BCL2*, and *ATG12*.

The tissue-specific abundance of miRNAs, their biological targets, and their efficient secretion into body fluids as disease advances all contribute to their potential as both biomarkers and therapeutic targets. In the tear fluid of 3-month-old *Apoe*-ko mice, only miR-146a showed significant upregulation. Over time, both amyloidogenic/angiogenic and inflammatory miRNAs increased substantially, suggesting elevated secretion into extracellular biofluids (Figure 2C). This pattern aligns with our previous findings in transgenic APP-PS1 mice, a preclinical model of AD (Wijesinghe et al., 2023b). Notably, miRNAs -146a and -15a were consistently upregulated in the neocortex-hippocampus, eye tissue, and tear fluid of 9-month-old *Apoe*-ko mice compared to controls. Increased secretion of these miRNAs has been reported in AD patient cerebrospinal fluid (CSF) (Lukiw et al., 2012; Sørensen et al., 2016). CSF drains into the lymphatic system via lymphatic ducts adjacent to the olfactory tract as it traverses the cribriform plate, while lymphatic drainage from the orbit and its associated structures remains under investigation (Machiele et al., 2024; Adigun and AI-Dhahir, 2023). However, the functional similarities between CSF and tear fluid suggest potential commonalities (Król-Grzymała et al., 2022). Thus, the upregulation of proinflammatory miR-146a and anti-amyloidogenic/angiogenic miR-15a in tear fluid may indicate their translational potential as non-invasive biomarkers for ApoE dysfunction.

Population-based studies consistently demonstrate that the *APOE4* allele is associated with a reduced risk of age-related macular degeneration (AMD, a retinal degenerative disease) (Xiying et al., 2017; Rasmussen et al., 2023), which is in contrast to its known association with increased risk for AD. Interestingly, our findings in 9-month-old *Apoe*-ko mice on high-fat versus regular diets shed light on *APOE4*’s role through miRNA dysregulation. On a high-fat diet, inflammatory miRNAs -125b, −146a, and −342 were significantly upregulated in both neocortex-hippocampus and eye tissues. Conversely, anti-amyloidogenic/angiogenic miRNAs −101a and −374c were significantly downregulated in the neocortex-hippocampus, indicating potential AD risk, while anti-amyloidogenic/angiogenic miRNAs −15a and −374c were significantly upregulated in the eye tissues, suggesting a protective effect in AMD. Notably, tear fluid miRNA levels showed no significant differences between high-fat and regular diet groups, as comparisons were limited to *Apoe*-ko strains. Previous studies typically use C57BL/6 J mice on a high-fat diet as controls for high-fat diet *Apoe*-ko mice (Cao et al., 2020).

APP is primarily associated with neurons but is also expressed in brain endothelial cells and astrocytes, though to a lesser extent (Hampel et al., 2021; Wang et al., 2021). We identified intraneuronal inclusions of APP/Aβ peptides in brain and retinal tissues, as well as in brain endothelial cells at the meninges (Figures 5A, 6A and Supplementary Figures S4, S6). Under normal homeostasis, APP is sequentially cleaved to produce Aβ peptides, which are cleared through various mechanisms, including phagocytosis by immune cells, transport across the blood–brain barrier (BBB), and interstitial fluid pathways like the glymphatic and perivascular drainages (O’Brien and Wong, 2011; Zuroff et al., 2017; Wijesinghe et al., 2023a). An imbalance between APP/Aβ peptide production and clearance results in the aggregation of neurotoxic oligomers and plaques (Hampel et al., 2021; Wang et al., 2021). Moreover, previous studies suggest that soluble Aβ species contribute significantly to cognitive impairment and synaptic toxicity in AD, acting earlier and independently of Aβ plaques and tau (Bloom, 2014; Koss et al., 2016; Hampel et al., 2021; Wang et al., 2021; Haynes et al., 2024).

We maintained consistent, high-resolution confocal microscopy settings for both negative controls and experimental samples, enabling clear comparisons of 6E10+ APP/Aβ peptide labeling between *Apoe*-ko and control mice in brain and retinal tissues, and their clearance over time relative to glial cell markers (Figures 5, 6, and Supplementary Figures S4–S7). The 6E10 antibody, which binds APP and Aβ at the 1–16 amino acid region, including plaques. Our previous study involving 5xFAD and C57BL/6 J mice demonstrated the presence of 6E10+ APP/Aβ peptides in neuroretina samples and observed treatment-induced changes (Wijesinghe et al., 2023a). Although developed for human APP/Aβ peptides, 6E10 also binds mouse APP/Aβ due to shared epitopes (Youmans et al., 2012; Hampel et al., 2021; Yamamoto et al., 2021; Wijesinghe et al., 2023a). This was confirmed using a knockout-validated anti-rabbit monoclonal APP antibody, verifying APP presence in the neocortex-hippocampus and retina of *Apoe*-ko and control mice (Supplementary Figures S4, S6). For further validation, we included a mouse monoclonal 12F4 antibody, targeting Aβ 1–42 amino acid residues. In brain tissue, 12F4 labeled only the endothelial cells in the meninges (Supplementary Figure S4J), while in the retina, it labeled the ILM, possibly reflecting soluble Aβ related to glymphatic clearance (Wijesinghe et al., 2023a) and an Aβ plaque-like deposit in the INL (Supplementary Figure S6D). Notably, despite being a mouse monoclonal antibody, 12F4 did not label any intraneuronal Aβ species.

The relationship between ApoE and Aβ clearance is complex, influenced by ApoE isoforms, lipidation status, and interactions with various proteins and receptors. ApoE binds to receptors like LRP1 (LDL receptor-related protein 1) at the BBB to aid in clearing soluble Aβ (Wang et al., 2021). It also acts as a ligand promoting microglial phagocytosis of insoluble Aβ. Regardless of the isoform, ApoE is recognized as a TREM2 ligand *in vitro*, potentially stimulating TREM2 functions (Huynh et al., 2017; Jendresen et al., 2017; Krasemann et al., 2017; Yeh et al., 2017). The TREM2-ApoE pathway is crucial for regulating microglial activity in neurodegenerative diseases and may help restore homeostatic microglia (Krasemann et al., 2017). ApoE deficiency impacts microglial recruitment to Aβ plaques, a phenotype similar to TREM2 deficiency (Ulrich et al., 2018; Zhao et al., 2018). In our study, cells expressing Trem2 were identified beyond colocalization with Iba1+ microglia/macrophages (Supplementary Figures S5, S7). This may result from soluble TREM2 binding to ligands on apoptotic neurons, facilitating TREM2-mediated phagocytosis (Hsieh et al., 2009). Additionally, compared to age-matched controls, 9-month-old *Apoe*-ko mice showed significantly higher levels of APP/A*β* peptides in the neocortex-hippocampus and retina, underscoring the essential role of ApoE in APP/Aβ clearance compared to Trem2.

Unlike transgenic models that replicate familial AD or overexpress pathological traits, we used *Apoe*-ko mice to study mechanisms relevant to human *APOE4* carriers, who are at higher risk for late-onset AD. Previous studies on aging human brains without a family history of AD showed significantly increased Aβ depositions in *APOE4* carriers (Wijesinghe et al., 2016; Hong et al., 2022). In this study, 9-month-old *Apoe*-ko mice, which resembles *APOE4* carriers (Janssen et al., 2016), exhibited higher APP/Aβ peptide levels, indicating impaired clearance, whereas matched control mice had lower levels, suggesting efficient clearance. The relationship between ApoE dysfunction, miRNA dysregulation, and AD-related pathology highlights complex genetic and molecular mechanisms. ApoE deficiency not only disrupts lipid metabolism, APP/Aβ peptide clearance, and glial homeostasis but also interacts with specific miRNAs, influencing disease progression and severity. These findings emphasize the critical role of ApoE and reveal miRNA biomarkers associated with ApoE dysfunction, paving the way for personalized treatments.

This study has some limitations, including a small sample size, a focus on female mice, and analysis at only two time points (3 and 9 months). We determined the sample size using the resource equation method to test our hypothesis. Since age, female sex, and genetic predispositions are non-modifiable AD risk factors, we focused on female mice. We limited our analysis to 3- and 9-month time points to investigate early changes in tear fluids as potential non-invasive biomarkers. Additionally, we compared *Apoe*-ko mice on a high-fat and regular diets to assess diet-based miRNA and mRNA dysregulations, but not protein levels.

## 5 Conclusion

Our study underscores ApoE’s role in AD pathogenesis through impaired glial homeostasis, potentially due to ApoE deficiency, increased APP/Aβ peptide accumulation, and disrupted lipid metabolism. The dysregulation of circulating inflammatory and amyloidogenic/angiogenic miRNAs in *Apoe*-ko mice at both 3-month-old and 9-month-old ages suggests the potential for developing tear-based biomarkers for individuals with the ApoE dysfunction.

## Data Availability

The original contributions presented in the study are included in the article/Supplementary material, further inquiries can be directed to the corresponding author.

## Supplementary material

SUPPLEMENTARY FIGURE S1 Relative mRNA expression level in the neocortex-hippocampus. Column graphs illustrate the mean 2^-∆Ct^ values across 3-month-old and 9-month-old *Apoe*-ko mice and controls. Pooled neocortex-hippocampus tissue samples (*n* = 4 per group) were used to determine relative mRNA levels. Differentially expressed target mRNAs are defined based on a 1.5-fold or greater intergroup difference and a significant *P* value at **P* < 0.05, ***P* < 0.01, ****P* < 0.001, and *****P* < 0.0001 (2-way ANOVA with Bonferroni corrected multiple comparisons test). Error bars indicate the standard error of the mean.

SUPPLEMENTARY FIGURE S2 Relative mRNA expression level in the eye tissue. Column graphs illustrate mean 2^-∆Ct^ value across 3-month-old and 9-month-old *Apoe*-ko mice and controls. Pooled eye tissue sample (*n* = 4 per group) was used to determine the relative mRNA level. Differentially expressed target mRNA is defined based on a 1.5-fold and above intergroup difference and a sigficant *P* value at **P* < 0.05, ***P* < 0.001, and *****P* < 0.0001 (2-way ANOVA with Bonferroni corrected multiple comparisons test). Error bars indicate the standard error of the mean.

SUPPLEMENTARY FIGURE S3 Relative mRNA expression level in the eye tissue of high fat diet *Apoe*-ko mice compared with regular diet *Apoe*-ko mice. Column graphs illustrate the mean 2^-∆Ct^ values between high-fat and regular diet 9-month-old *Apoe*-ko mice. Pooled eye tissue samples (*n* = 4-5 per group) were used to determine relative mRNA levels. Differentially expressed target mRNA is defined based on a 1.5-fold and above intergroup difference and a significant *P* value at ***P* < 0.001, and ****P* < 0.001 (Unpaired t-test, 2-tailed). Error bars indicate the standard error of the mean. (HFD- high fat diet; RD- regular diet).

SUPPLEMENTARY FIGURE S4 Differences in APP/Aβ peptide and astroglia expression in the neocortex-hippocampus of *Apoe*-ko and control mice. **(A)** Negative control demonstrating the absence of APP/Aβ peptides and astroglia in the hippocampal and neocortical regions. Higher magnification images depict 6E10+ APP/Aβ peptides in the **(B)** CA region (white dashed box), **(F)** neocortex (white arrowheads), and **(H)** meninges (red arrowheads). White dashed circles indicate 6E10-negative cells. **(C,G,I)** APP presence was verified using a rabbit monoclonal APP antibody. **(D,J)** A mouse monoclonal antibody, 12F4, specific to the 1-42 amino acid residues of Aβ peptides, showed positive signals only in the meninges (red arrowheads). **(E,K)** Expression patterns of 6E10+ APP/Aβ peptides in the hippocampal CA1 region and neocortex of 3-month-old and 9-month-old *Apoe*-ko mice and controls. (Scale bar: 20 μm).

SUPPLEMENTARY FIGURE S5 Trem2 expression with and without microglial colocalization in the neocortex and hippocampus of *Apoe*-ko and control mice. **(A–C)** Colocalization of microglia expressing Trem2 receptors (yellow arrowheads) and cells expressing only Trem2 (white dashed circles) in the hippocampal dentate gyrus, CA1 region, and neocortex. **(D,E)** Expression patterns of Trem2 and Iba1+ microglia in *Apoe*-ko and control mice in the CA1 region of the hippocampus and the neocortex, respectively. White dashed circles (in green channel) indicate cells expressing only Trem2, while yellow arrowheads indicate Iba1+ microglia expressing Trem2 (in both green and red channels). (Scale bar: 20 μm).

SUPPLEMENTARY FIGURE S6 Differences in APP/Aβ peptide and astroglial expression in the retina of *Apoe*-ko and control mice. **(A)** Negative control demonstrating the absence of APP/Aβ peptides and astroglia across retinal layers. **(B)** Higher magnification images showing 6E10+ APP/Aβ peptides predominantly within the GCL. **(C)** The presence of 6E10+ APP was confirmed using a rabbit monoclonal APP antibody in the GCL. A white dashed circle highlights diffuse deposition within the GCL in panels **(B,C)**. **(D)** Additionally, 12F4 showed positive signals at the inner limiting membrane (ILM). The white dashed circle indicates a 12F4+ Aβ plaque-like deposition within the INL in panel D. **(E,F)** Expression patterns of 6E10+ APP/Aβ peptides within the central and peripheral retinas of 3-month-old and 9-month-old *Apoe*-ko mice and controls. White dashed boxes highlight the localization of 6E10+ APP/Aβ peptides within the GCL in both central and peripheral retinas. (Scale bar: 20 μm) (NFL, nerve fiber layer; GCL, ganglion cell layer; IPL, inner plexiform layer; INL, inner nuclear layer; OPL, outer plexiform layer; ONL, outer nuclear layer).

SUPPLEMENTARY FIGURE S7 Trem2 expression with and without microglia/macrophage colocalization across the retinal layers of *Apoe*-ko and control mice. **(A,B)** Expression patterns of Trem2 and Iba1+ microglia/macrophages in *Apoe*-ko and control mice are shown for the central and peripheral retinas, respectively. White dashed boxes indicate retinal layers expressing Trem2, while yellow arrowheads indicate Iba1+ microglia/macrophages within the retinal layers. Trem2 and Iba1+ microglia/macrophage expression, with or without colocalization, was predominantly identified in the NFL-GCL, followed by the OPL. (Scale bar: 20 μm) (NFL: nerve fiber layer; GCL: ganglion cell layer; IPL: inner plexiform layer; INL: inner nuclear layer; OPL: outer plexiform layer; ONL: outer nuclear layer).

## References

[ref1] Adigun O. O. Al-Dhahir M. A. (2023). Anatomy, Head and Neck: Cerebrospinal Fluid. In: StatPearls. Treasure Island (FL): StatPearls Publishing. 29083815

[ref2] Agarwal V. Bell G. W. Nam J.-W. Bartel D. P. (2015). Predicting effective microRNA target sites in mammalian mRNAs. eLife 4:e05005. doi: 10.7554/eLife.05005, PMID: 26267216 PMC4532895

[ref3] Ansari A. Maffioletti E. Milanesi E. Marizzoni M. Frisoni G. B. Blin O.. (2019). miR-146a and miR-181a are involved in the progression of mild cognitive impairment to Alzheimer’s disease. Neurobiol. Aging 82, 102–109. doi: 10.1016/j.neurobiolaging.2019.06.005, PMID: 31437718

[ref4] Bali J. Gheinani A. H. Zurbriggen S. Rajendran L. (2012). Role of genes linked to sporadic Alzheimer’s disease risk in the production of β-amyloid peptides. Proc. Natl. Acad. Sci. USA 109, 15307–15311. doi: 10.1073/pnas.1201632109, PMID: 22949636 PMC3458335

[ref5] Bhattacharjee S. Zhao Y. Dua P. Rogaev E. I. Lukiw W. J. (2016). microRNA-34a-mediated Down-regulation of the microglial-enriched triggering receptor and phagocytosis-sensor TREM2 in age-related macular degeneration. PLoS One 11:e0150211. doi: 10.1371/journal.pone.0150211, PMID: 26949937 PMC4780721

[ref6] Bloom G. S. (2014). Amyloid-β and tau: the trigger and bullet in Alzheimer disease pathogenesis. JAMA Neurol. 71, 505–508. doi: 10.1001/jamaneurol.2013.5847 24493463 PMC12908160

[ref7] Cao X. Guo Y. Wang Y. Wang H. Liu D. Gong Y.. (2020). Effects of high-fat diet and Apoe deficiency on retinal structure and function in mice. Sci. Rep. 10:18601. doi: 10.1038/s41598-020-75576-7, PMID: 33139746 PMC7606505

[ref8] Cao J. Huang M. Guo L. Zhu L. Hou J. Zhang L.. (2021). MicroRNA-195 rescues ApoE4-induced cognitive deficits and lysosomal defects in Alzheimer’s disease pathogenesis. Mol. Psychiatry 26, 4687–4701. doi: 10.1038/s41380-020-0824-3, PMID: 32632205 PMC7785685

[ref9] Charan J. Kantharia N. D. (2013). How to calculate sample size in animal studies? J. Pharmacol. Pharmacother. 4, 303–306. doi: 10.4103/0976-500X.119726, PMID: 24250214 PMC3826013

[ref10] Chen H.-K. Liu Z. Meyer-Franke A. Brodbeck J. Miranda R. D. McGuire J. G.. (2012). Small molecule structure correctors abolish detrimental effects of apolipoprotein E4 in cultured neurons. J. Biol. Chem. 287, 5253–5266. doi: 10.1074/jbc.M111.276162, PMID: 22158868 PMC3285306

[ref11] Culleton S. Niu M. Alexander M. McNally J. S. Yuan C. Parker D.. (2023). Extracranial carotid artery atherosclerotic plaque and APOE polymorphisms: a systematic review and meta-analysis. Front. Cardiovasc. Med. 10:1155916. doi: 10.3389/fcvm.2023.1155916, PMID: 38034385 PMC10683092

[ref12] De León H. Boué S. Schlage W. K. Boukharov N. Westra J. W. Gebel S.. (2014). A vascular biology network model focused on inflammatory processes to investigate atherogenesis and plaque instability. J. Transl. Med. 12:185. doi: 10.1186/1479-5876-12-185, PMID: 24965703 PMC4227037

[ref13] DeRosa B. A. Simon S. A. Velez C. A. Vance J. M. Pericak-Vance M. A. Dykxhoorn D. M. (2022). Generation of two iPSC lines (UMi038-a & UMi039-a) from siblings bearing an Alzheimer’s disease-associated variant in SORL1. Stem Cell Res. 62:102823. doi: 10.1016/j.scr.2022.102823, PMID: 35671596

[ref14] Fan W. Liang C. Ou M. Zou T. Sun F. Zhou H.. (2020). MicroRNA-146a is a wide-reaching Neuroinflammatory regulator and potential treatment target in neurological diseases. Front. Mol. Neurosci. 13:90. doi: 10.3389/fnmol.2020.00090, PMID: 32581706 PMC7291868

[ref15] Faraji P. Kühn H. Ahmadian S. (2024). Multiple roles of Apolipoprotein E4 in oxidative lipid metabolism and Ferroptosis during the pathogenesis of Alzheimer’s disease. J. Mol. Neurosci. 74:62. doi: 10.1007/s12031-024-02224-4 38958788 PMC11222241

[ref16] Fitz N. F. Tapias V. Cronican A. A. Castranio E. L. Saleem M. Carter A. Y.. (2015). Opposing effects of Apoe/Apoa1 double deletion on amyloid-β pathology and cognitive performance in APP mice. Brain 138, 3699–3715. doi: 10.1093/brain/awv293 26510953 PMC4731410

[ref17] Friedman R. C. Farh K. K.-H. Burge C. B. Bartel D. P. (2009). Most mammalian mRNAs are conserved targets of microRNAs. Genome Res. 19, 92–105. doi: 10.1101/gr.082701.108, PMID: 18955434 PMC2612969

[ref18] Fu Y. Hu X. Zheng C. Sun G. Xu J. Luo S.. (2019). Intrahippocampal miR-342-3p inhibition reduces β-amyloid plaques and ameliorates learning and memory in Alzheimer’s disease. Metab. Brain Dis. 34, 1355–1363. doi: 10.1007/s11011-019-00438-9 31134481

[ref19] Fu W. Ye Y. Hu F. (2024). LncRNA XIST promotes neovascularization in diabetic retinopathy by regulating miR-101-3p/VEGFA. Arch. Endocrinol. Metab. 68:e230097. doi: 10.20945/2359-4292-2023-0097, PMID: 38739522 PMC11156180

[ref20] Fuentes D. Fernández N. García Y. García T. Morales A. R. Menéndez R. (2018). Age-related changes in the behavior of Apolipoprotein E knockout mice. Behav. Sci. 8:33. doi: 10.3390/bs8030033, PMID: 29510495 PMC5867486

[ref21] Gaire B. P. Koronyo Y. Fuchs D.-T. Shi H. Rentsendorj A. Danziger R.. (2024). Alzheimer’s disease pathophysiology in the retina. Prog. Retin. Eye Res. 101:101273. doi: 10.1016/j.preteyeres.2024.101273, PMID: 38759947 PMC11285518

[ref22] Grubman A. Chew G. Ouyang J. F. Sun G. Choo X. Y. McLean C.. (2019). A single-cell atlas of entorhinal cortex from individuals with Alzheimer’s disease reveals cell-type-specific gene expression regulation. Nat. Neurosci. 22, 2087–2097. doi: 10.1038/s41593-019-0539-4, PMID: 31768052

[ref23] Guerreiro R. Brás J. Hardy J. (2013). SnapShot: genetics of Alzheimer’s disease. Cell 155, 968–968.e1. doi: 10.1016/j.cell.2013.10.037 24209629

[ref24] Hampel H. Hardy J. Blennow K. Chen C. Perry G. Kim S. H.. (2021). The amyloid-β pathway in Alzheimer’s disease. Mol. Psychiatry 26, 5481–5503. doi: 10.1038/s41380-021-01249-0, PMID: 34456336 PMC8758495

[ref25] Hart de Ruyter F. J. Evers M. J. A. P. Morrema T. H. J. Dijkstra A. A. den Haan J. Twisk J. W. R.. (2024). Neuropathological hallmarks in the post-mortem retina of neurodegenerative diseases. Acta Neuropathol. 148:24. doi: 10.1007/s00401-024-02769-z, PMID: 39160362 PMC11333524

[ref26] Haynes J. R. Whitmore C. A. Behof W. J. Landman C. A. Ong H. H. Feld A. P.. (2024). Targeting soluble amyloid-beta oligomers with a novel nanobody. Sci. Rep. 14:16086. doi: 10.1038/s41598-024-66970-6 38992064 PMC11239946

[ref27] Hong Y. J. Kim C.-M. Lee J. H. Sepulcre J. (2022). Correlations between APOE4 allele and regional amyloid and tau burdens in cognitively normal older individuals. Sci. Rep. 12:14307. doi: 10.1038/s41598-022-18325-2, PMID: 35995824 PMC9395408

[ref28] Hsieh C. L. Koike M. Spusta S. C. Niemi E. C. Yenari M. Nakamura M. C.. (2009). A role for TREM2 ligands in the phagocytosis of apoptotic neuronal cells by microglia. J. Neurochem. 109, 1144–1156. doi: 10.1111/j.1471-4159.2009.06042.x, PMID: 19302484 PMC3087597

[ref29] Huang H.-Y. Lin Y.-C.-D. Cui S. Huang Y. Tang Y. Xu J.. (2022). miRTarBase update 2022: an informative resource for experimentally validated miRNA-target interactions. Nucleic Acids Res. 50, D222–D230. doi: 10.1093/nar/gkab1079, PMID: 34850920 PMC8728135

[ref30] Huynh T.-P. V. Davis A. A. Ulrich J. D. Holtzman D. M. (2017). Apolipoprotein E and Alzheimer’s disease: the influence of apolipoprotein E on amyloid-β and other amyloidogenic proteins. J. Lipid Res. 58, 824–836. doi: 10.1194/jlr.R075481, PMID: 28246336 PMC5408619

[ref31] Janssen C. I. F. Jansen D. Mutsaers M. P. C. Dederen P. J. W. C. Geenen B. Mulder M. T.. (2016). The effect of a high-fat diet on brain plasticity, inflammation and cognition in female ApoE4-Knockin and ApoE-knockout mice. PLoS One 11:e0155307. doi: 10.1371/journal.pone.0155307, PMID: 27171180 PMC4865084

[ref32] Jendresen C. Årskog V. Daws M. R. Nilsson L. N. G. (2017). The Alzheimer’s disease risk factors apolipoprotein E and TREM2 are linked in a receptor signaling pathway. J. Neuroinflammation 14:59. doi: 10.1186/s12974-017-0835-4, PMID: 28320424 PMC5360024

[ref33] Kehl T. Kern F. Backes C. Fehlmann T. Stöckel D. Meese E.. (2020). miRPathDB 2.0: a novel release of the miRNA pathway dictionary database. Nucleic Acids Res. 48, D142–D147. doi: 10.1093/nar/gkz1022, PMID: 31691816 PMC7145528

[ref34] Kim J. Basak J. M. Holtzman D. M. (2009). The role of apolipoprotein E in Alzheimer’s disease. Neuron 63, 287–303. doi: 10.1016/j.neuron.2009.06.026, PMID: 19679070 PMC3044446

[ref35] Komai M. Noda Y. Ikeda A. Kaneshiro N. Kamikubo Y. Sakurai T.. (2024). Nuclear SphK2/S1P signaling is a key regulator of ApoE production and Aβ uptake in astrocytes. J. Lipid Res. 65:100510. doi: 10.1016/j.jlr.2024.100510, PMID: 38280459 PMC10907773

[ref36] Koss D. J. Jones G. Cranston A. Gardner H. Kanaan N. M. Platt B. (2016). Soluble pre-fibrillar tau and β-amyloid species emerge in early human Alzheimer’s disease and track disease progression and cognitive decline. Acta Neuropathol. 132, 875–895. doi: 10.1007/s00401-016-1632-3, PMID: 27770234 PMC5106509

[ref37] Krasemann S. Madore C. Cialic R. Baufeld C. Calcagno N. El Fatimy R.. (2017). The TREM2-APOE pathway drives the transcriptional phenotype of dysfunctional microglia in neurodegenerative diseases. Immunity 47, 566–581.e9. doi: 10.1016/j.immuni.2017.08.008, PMID: 28930663 PMC5719893

[ref38] Król-Grzymała A. Sienkiewicz-Szłapka E. Fiedorowicz E. Rozmus D. Cieślińska A. Grzybowski A. (2022). Tear biomarkers in Alzheimer’s and Parkinson’s diseases, and multiple sclerosis: implications for diagnosis (systematic review). Int. J. Mol. Sci. 23:10123. doi: 10.3390/ijms231710123, PMID: 36077520 PMC9456033

[ref39] Lane-Donovan C. Wong W. M. Durakoglugil M. S. Wasser C. R. Jiang S. Xian X.. (2016). Genetic restoration of plasma ApoE improves cognition and partially restores synaptic defects in ApoE-deficient mice. J. Neurosci. 36, 10141–10150. doi: 10.1523/JNEUROSCI.1054-16.2016, PMID: 27683909 PMC5039258

[ref40] Lee S. Jiang K. McIlmoyle B. To E. Xu Q. A. Hirsch-Reinshagen V.. (2020). Amyloid Beta Immunoreactivity in the retinal ganglion cell layer of the Alzheimer’s eye. Front. Neurosci. 14:758. doi: 10.3389/fnins.2020.00758, PMID: 32848548 PMC7412634

[ref41] Liu H.-Y. Fu X. Li Y.-F. Li X.-L. Ma Z.-Y. Zhang Y.. (2019). miR-15b-5p targeting amyloid precursor protein is involved in the anti-amyloid eflect of curcumin in swAPP695-HEK293 cells. Neural Regen. Res. 14, 1603–1609. doi: 10.4103/1673-5374.255979, PMID: 31089060 PMC6557094

[ref42] Liu C.-C. Liu C.-C. Kanekiyo T. Xu H. Bu G. (2013). Apolipoprotein E and Alzheimer disease: risk, mechanisms and therapy. Nat. Rev. Neurol. 9, 106–118. doi: 10.1038/nrneurol.2012.263, PMID: 23296339 PMC3726719

[ref43] Liu L. Xu J. Huang X. Wang Y. Ma X. Wang X.. (2024). DHA dietary intervention caused different hippocampal lipid and protein profile in ApoE−/− and C57BL/6J mice. Biomed. Pharmacother. 177:117088. doi: 10.1016/j.biopha.2024.117088, PMID: 38971007

[ref44] Lo Sasso G. Schlage W. K. Boué S. Veljkovic E. Peitsch M. C. Hoeng J. (2016). The Apoe(−/−) mouse model: a suitable model to study cardiovascular and respiratory diseases in the context of cigarette smoke exposure and harm reduction. J. Transl. Med. 14:146. doi: 10.1186/s12967-016-0901-1, PMID: 27207171 PMC4875735

[ref45] Long J. M. Lahiri D. K. (2011). MicroRNA-101 downregulates Alzheimer’s amyloid-β precursor protein levels in human cell cultures and is differentially expressed. Biochem. Biophys. Res. Commun. 404, 889–895. doi: 10.1016/j.bbrc.2010.12.053, PMID: 21172309 PMC3372402

[ref46] Lukiw W. J. Alexandrov P. N. Zhao Y. Hill J. M. Bhattacharjee S. (2012). Spreading of Alzheimer’s disease inflammatory signaling through soluble micro-RNA. Neuroreport 23, 621–626. doi: 10.1097/WNR.0b013e32835542b0 22660168 PMC4467540

[ref47] Machiele R. Lopez M. J. Czyz C. N. (2024). Anatomy, head and neck: eye lacrimal gland. Treasure Island, FL: StatPearls Publishing. 30422509

[ref48] Mathys H. Davila-Velderrain J. Peng Z. Gao F. Mohammadi S. Young J. Z.. (2019). Single-cell transcriptomic analysis of Alzheimer’s disease. Nature 570, 332–337. doi: 10.1038/s41586-019-1195-2, PMID: 31042697 PMC6865822

[ref49] Murgia N. Ma Y. Najam S. S. Liu Y. Przybys J. Guo C.. (2022). In vivo reductionist approach identifies miR-15a protecting mice from obesity. Front. Endocrinol. 13:867929. doi: 10.3389/fendo.2022.867929, PMID: 35873003 PMC9302447

[ref50] Narasimhan S. Holtzman D. M. Apostolova L. G. Cruchaga C. Masters C. L. Hardy J.. (2024). Apolipoprotein E in Alzheimer’s disease trajectories and the next-generation clinical care pathway. Nat. Neurosci. 27, 1236–1252. doi: 10.1038/s41593-024-01669-5, PMID: 38898183

[ref51] O’Brien R. J. Wong P. C. (2011). Amyloid precursor protein processing and Alzheimer’s disease. Annu. Rev. Neurosci. 34, 185–204. doi: 10.1146/annurev-neuro-061010-113613, PMID: 21456963 PMC3174086

[ref52] Parhizkar S. Holtzman D. M. (2022). APOE mediated neuroinflammation and neurodegeneration in Alzheimer’s disease. Semin. Immunol. 59:101594. doi: 10.1016/j.smim.2022.101594, PMID: 35232622 PMC9411266

[ref53] Piedrahita J. A. Zhang S. H. Hagaman J. R. Oliver P. M. Maeda N. (1992). Generation of mice carrying a mutant apolipoprotein E gene inactivated by gene targeting in embryonic stem cells. Proc. Natl. Acad. Sci. USA 89, 4471–4475. doi: 10.1073/pnas.89.10.4471, PMID: 1584779 PMC49104

[ref54] Pogue A. I. Lukiw W. J. (2018). Up-regulated pro-inflammatory MicroRNAs (miRNAs) in Alzheimer’s disease (AD) and age-related macular degeneration (AMD). Cell. Mol. Neurobiol. 38, 1021–1031. doi: 10.1007/s10571-017-0572-3, PMID: 29302837 PMC11481951

[ref55] Rasmussen K. L. Tybjærg-Hansen A. Nordestgaard B. G. Frikke-Schmidt R. (2023). Associations of Alzheimer disease-protective APOE variants with age-related macular degeneration. JAMA Ophthalmol. 141, 13–21. doi: 10.1001/jamaophthalmol.2022.4602, PMID: 36394841 PMC9673029

[ref56] Raulin A.-C. Doss S. V. Trottier Z. A. Ikezu T. C. Bu G. Liu C.-C. (2022). ApoE in Alzheimer’s disease: pathophysiology and therapeutic strategies. Mol. Neurodegener. 17:72. doi: 10.1186/s13024-022-00574-4, PMID: 36348357 PMC9644639

[ref57] Rogaeva E. Meng Y. Lee J. H. Gu Y. Kawarai T. Zou F.. (2007). The neuronal sortilin-related receptor SORL1 is genetically associated with Alzheimer disease. Nat. Genet. 39, 168–177. doi: 10.1038/ng1943, PMID: 17220890 PMC2657343

[ref58] Safieh M. Korczyn A. D. Michaelson D. M. (2019). ApoE4: an emerging therapeutic target for Alzheimer’s disease. BMC Med. 17:64. doi: 10.1186/s12916-019-1299-4, PMID: 30890171 PMC6425600

[ref59] Schmittgen T. D. Livak K. J. (2008). Analyzing real-time PCR data by the comparative C(T) method. Nat. Protoc. 3, 1101–1108. doi: 10.1038/nprot.2008.73 18546601

[ref60] Shannon P. Markiel A. Ozier O. Baliga N. S. Wang J. T. Ramage D.. (2003). Cytoscape: a software environment for integrated models of biomolecular interaction networks. Genome Res. 13, 2498–2504. doi: 10.1101/gr.1239303, PMID: 14597658 PMC403769

[ref61] Shao N.-Y. Hu H. Y. Yan Z. Xu Y. Hu H. Menzel C.. (2010). Comprehensive survey of human brain microRNA by deep sequencing. BMC Genomics 11:409. doi: 10.1186/1471-2164-11-409, PMID: 20591156 PMC2996937

[ref62] Sherman B. T. Hao M. Qiu J. Jiao X. Baseler M. W. Lane H. C.. (2022). DAVID: a web server for functional enrichment analysis and functional annotation of gene lists (2021 update). Nucleic Acids Res. 50, W216–W221. doi: 10.1093/nar/gkac194, PMID: 35325185 PMC9252805

[ref63] Sidiqi A. Wahl D. Lee S. Ma D. To E. Cui J.. (2020). In vivo retinal fluorescence imaging with curcumin in an Alzheimer mouse model. Front. Neurosci. 14:713. doi: 10.3389/fnins.2020.00713, PMID: 32719582 PMC7350785

[ref64] Sørensen S. S. Nygaard A.-B. Christensen T. (2016). miRNA expression profiles in cerebrospinal fluid and blood of patients with Alzheimer’s disease and other types of dementia - an exploratory study. Transl. Neurodegener. 5:6. doi: 10.1186/s40035-016-0053-5, PMID: 26981236 PMC4791887

[ref65] Stapleton M. C. Koch S. P. Cortes D. R. E. Wyman S. Schwab K. E. Mueller S.. (2023). Apolipoprotein-E deficiency leads to brain network alteration characterized by diffusion MRI and graph theory. Front. Neurosci. 17:1183312. doi: 10.3389/fnins.2023.1183312, PMID: 38075287 PMC10702609

[ref66] Sun P. Ma F. Xu Y. Zhou C. Stetler R. A. Yin K.-J. (2021). Genetic deletion of endothelial microRNA-15a/16-1 promotes cerebral angiogenesis and neurological recovery in ischemic stroke through Src signaling pathway. J. Cereb. Blood Flow Metab. 41, 2725–2742. doi: 10.1177/0271678X211010351, PMID: 33910400 PMC8504951

[ref67] Tamminen M. Mottino G. Qiao J. H. Breslow J. L. Frank J. S. (1999). Ultrastructure of early lipid accumulation in ApoE-deficient mice. Arterioscler. Thromb. Vasc. Biol. 19, 847–853. doi: 10.1161/01.atv.19.4.847, PMID: 10195908

[ref68] Ulrich J. D. Ulland T. K. Mahan T. E. Nyström S. Nilsson K. P. Song W. M.. (2018). ApoE facilitates the microglial response to amyloid plaque pathology. J. Exp. Med. 215, 1047–1058. doi: 10.1084/jem.20171265 29483128 PMC5881464

[ref69] Untergasser A. Nijveen H. Rao X. Bisseling T. Geurts R. Leunissen J. A. M. (2007). Primer3Plus, an enhanced web interface to Primer3. Nucleic Acids Res. 35, W71–W74. doi: 10.1093/nar/gkm306, PMID: 17485472 PMC1933133

[ref70] Vilardo E. Barbato C. Ciotti M. Cogoni C. Ruberti F. (2010). MicroRNA-101 regulates amyloid precursor protein expression in hippocampal neurons. J. Biol. Chem. 285, 18344–18351. doi: 10.1074/jbc.M110.112664, PMID: 20395292 PMC2881760

[ref71] von Holt K. Lebrun S. Stinn W. Conroy L. Wallerath T. Schleef R. (2009). Progression of atherosclerosis in the Apo E−/− model: 12-month exposure to cigarette mainstream smoke combined with high-cholesterol/fat diet. Atherosclerosis 205, 135–143. doi: 10.1016/j.atherosclerosis.2008.11.031 19144336

[ref72] Wang D. Chen F. Han Z. Yin Z. Ge X. Lei P. (2021). Relationship between amyloid-β deposition and blood-brain barrier dysfunction in Alzheimer’s disease. Front. Cell. Neurosci. 15:695479. doi: 10.3389/fncel.2021.695479, PMID: 34349624 PMC8326917

[ref73] Wang C. Lu J. Sha X. Qiu Y. Chen H. Yu Z. (2023). TRPV1 regulates ApoE4-disrupted intracellular lipid homeostasis and decreases synaptic phagocytosis by microglia. Exp. Mol. Med. 55, 347–363. doi: 10.1038/s12276-023-00935-z, PMID: 36720919 PMC9981624

[ref74] Wang L.-L. Min L. Guo Q.-D. Zhang J.-X. Jiang H.-L. Shao S.. (2017). Profiling microRNA from brain by microarray in a transgenic mouse model of Alzheimer’s disease. Biomed. Res. Int. 2017, 8030369–8030311. doi: 10.1155/2017/8030369, PMID: 29057267 PMC5625804

[ref75] Wijesinghe P. Shankar S. K. Yasha T. C. Gorrie C. Amaratunga D. Hulathduwa S.. (2016). Vascular contributions in Alzheimer’s disease-related neuropathological changes: first autopsy evidence from a south Asian aging population. J. Alzheimers Dis. 54, 1607–1618. doi: 10.3233/JAD-160425 27589527

[ref76] Wijesinghe P. Steinbusch H. W. M. Shankar S. K. Yasha T. C. De Silva K. R. D. (2020). Circle of Willis abnormalities and their clinical importance in ageing brains: a cadaveric anatomical and pathological study. J. Chem. Neuroanat. 106:101772. doi: 10.1016/j.jchemneu.2020.101772, PMID: 32165168

[ref77] Wijesinghe P. Whitmore C. A. Campbell M. Li C. Tsuyuki M. To E.. (2023a). Ergothioneine, a dietary antioxidant improves amyloid beta clearance in the neuroretina of a mouse model of Alzheimer’s disease. Front. Neurosci. 17:1107436. doi: 10.3389/fnins.2023.1107436, PMID: 36998724 PMC10043244

[ref78] Wijesinghe P. Xi J. Cui J. Campbell M. Pham W. Matsubara J. A. (2023b). MicroRNAs in tear fluids predict underlying molecular changes associated with Alzheimer’s disease. Life Sci Alliance 6:e202201757. doi: 10.26508/lsa.202201757, PMID: 36941055 PMC10027899

[ref79] Xiying M. Wenbo W. Wangyi F. Qinghuai L. (2017). Association of Apolipoprotein E Polymorphisms with age-related macular degeneration subtypes: An updated systematic review and Meta-analysis. Arch. Med. Res. 48, 370–377. doi: 10.1016/j.arcmed.2017.08.002, PMID: 28889998

[ref80] Xu Q. A. Boerkoel P. Hirsch-Reinshagen V. Mackenzie I. R. Hsiung G.-Y. R. Charm G.. (2022). Müller cell degeneration and microglial dysfunction in the Alzheimer’s retina. Acta Neuropathol. Commun. 10:145. doi: 10.1186/s40478-022-01448-y, PMID: 36199154 PMC9533552

[ref81] Yamamoto K. Yamamoto R. Kato N. (2021). Amyloid β and amyloid precursor protein synergistically suppress large-conductance calcium-activated Potassium Channel in cortical neurons. Front. Aging Neurosci. 13:660319. doi: 10.3389/fnagi.2021.660319, PMID: 34149396 PMC8211014

[ref82] Yamazaki Y. Zhao N. Caulfield T. R. Liu C.-C. Bu G. (2019). Apolipoprotein E and Alzheimer disease: pathobiology and targeting strategies. Nat. Rev. Neurol. 15, 501–518. doi: 10.1038/s41582-019-0228-7, PMID: 31367008 PMC7055192

[ref83] Yang L. G. March Z. M. Stephenson R. A. Narayan P. S. (2023). Apolipoprotein E in lipid metabolism and neurodegenerative disease. Trends Endocrinol. Metab. 34, 430–445. doi: 10.1016/j.tem.2023.05.002, PMID: 37357100 PMC10365028

[ref84] Yeh F. L. Hansen D. V. Sheng M. (2017). TREM2, microglia, and neurodegenerative diseases. Trends Mol. Med. 23, 512–533. doi: 10.1016/j.molmed.2017.03.008 28442216

[ref85] Youmans K. L. Tai L. M. Kanekiyo T. Stine W. B. Michon S.-C. Nwabuisi-Heath E.. (2012). Intraneuronal Aβ detection in 5xFAD mice by a new Aβ-specific antibody. Mol. Neurodegener. 7:8. doi: 10.1186/1750-1326-7-8, PMID: 22423893 PMC3355009

[ref86] Zhang N. Li W.-W. Lv C.-M. Gao Y.-W. Liu X.-L. Zhao L. (2020). miR-16-5p and miR-19b-3p prevent amyloid β-induced injury by targeting BACE1 in SH-SY5Y cells. Neuroreport 31, 205–212. doi: 10.1097/WNR.0000000000001379, PMID: 31876684

[ref87] Zhang J. Liu Q. (2015). Cholesterol metabolism and homeostasis in the brain. Protein Cell 6, 254–264. doi: 10.1007/s13238-014-0131-3, PMID: 25682154 PMC4383754

[ref88] Zhao Y. Jaber V. Lukiw W. J. (2016). Over-expressed pathogenic miRNAs in Alzheimer’s disease (AD) and prion disease (PrD) drive deficits in TREM2-mediated Aβ42 peptide clearance. Front. Aging Neurosci. 8:140. doi: 10.3389/fnagi.2016.00140, PMID: 27378912 PMC4906923

[ref89] Zhao L. Li J. (2024). Microglial uptake of hADSCs-Exo mitigates neuroinflammation in ICH. Cell. Signal. 119:111146. doi: 10.1016/j.cellsig.2024.111146, PMID: 38499232

[ref90] Zhao Y. Wu X. Li X. Jiang L.-L. Gui X. Liu Y.. (2018). TREM2 is a receptor for β-amyloid that mediates microglial function. Neuron 97, 1023–1031.e7. doi: 10.1016/j.neuron.2018.01.031, PMID: 29518356 PMC5889092

[ref91] Zheng S. Zhang K. Zhang Y. He J. Ouyang Y. Lang R.. (2023). Human umbilical cord mesenchymal stem cells inhibit Pyroptosis of renal tubular epithelial cells through miR-342-3p/Caspase1 signaling pathway in diabetic nephropathy. Stem Cells Int. 2023, 5584894–5584812. doi: 10.1155/2023/5584894, PMID: 37056456 PMC10089783

[ref92] Zuroff L. Daley D. Black K. L. Koronyo-Hamaoui M. (2017). Clearance of cerebral Aβ in Alzheimer’s disease: reassessing the role of microglia and monocytes. Cell. Mol. Life Sci. 74, 2167–2201. doi: 10.1007/s00018-017-2463-7, PMID: 28197669 PMC5425508

