# Supplementary figures and images for: Apolipoprotein E dysfunction in Alzheimer’s disease: a study on miRNA regulation, glial markers, and amyloid pathology
Source: Front Aging Neurosci. 2024 Dec 18;16:1495615. doi: 10.3389/fnagi.2024.1495615 (PMC11688329; doi:10.3389/fnagi.2024.1495615)

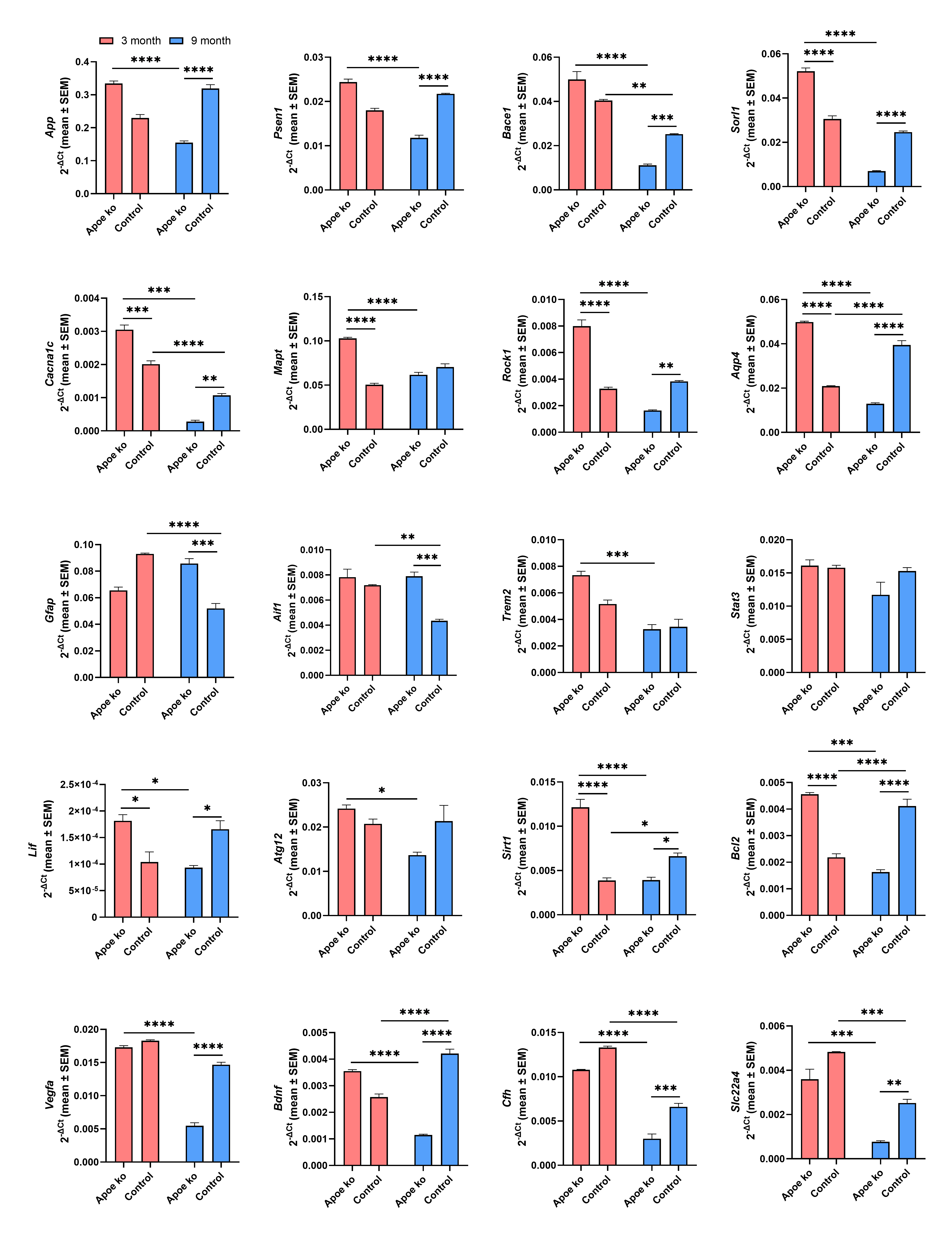

Supplement: SUPPLEMENTARY FIGURE S1 — Relative mRNA expression level in the neocortex-hippocampus. Column graphs illustrate the mean 2-∆Ct values across 3-month-old and 9-month-old Apoe-ko mice and controls. Pooled neocortex-hippocampus tissue samples (n = 4 per group) were used to determine relative mRNA levels. Differentially expressed target mRNAs are defined based on a 1.5-fold or greater intergroup difference and a significant P value at *P < 0.05, **P < 0.01, ***P < 0.001, and ****P < 0.0001 (2-way ANOVA with Bonferroni corrected multiple comparisons test). Error bars indicate the standard error of the mean. [file Image_1.tif]

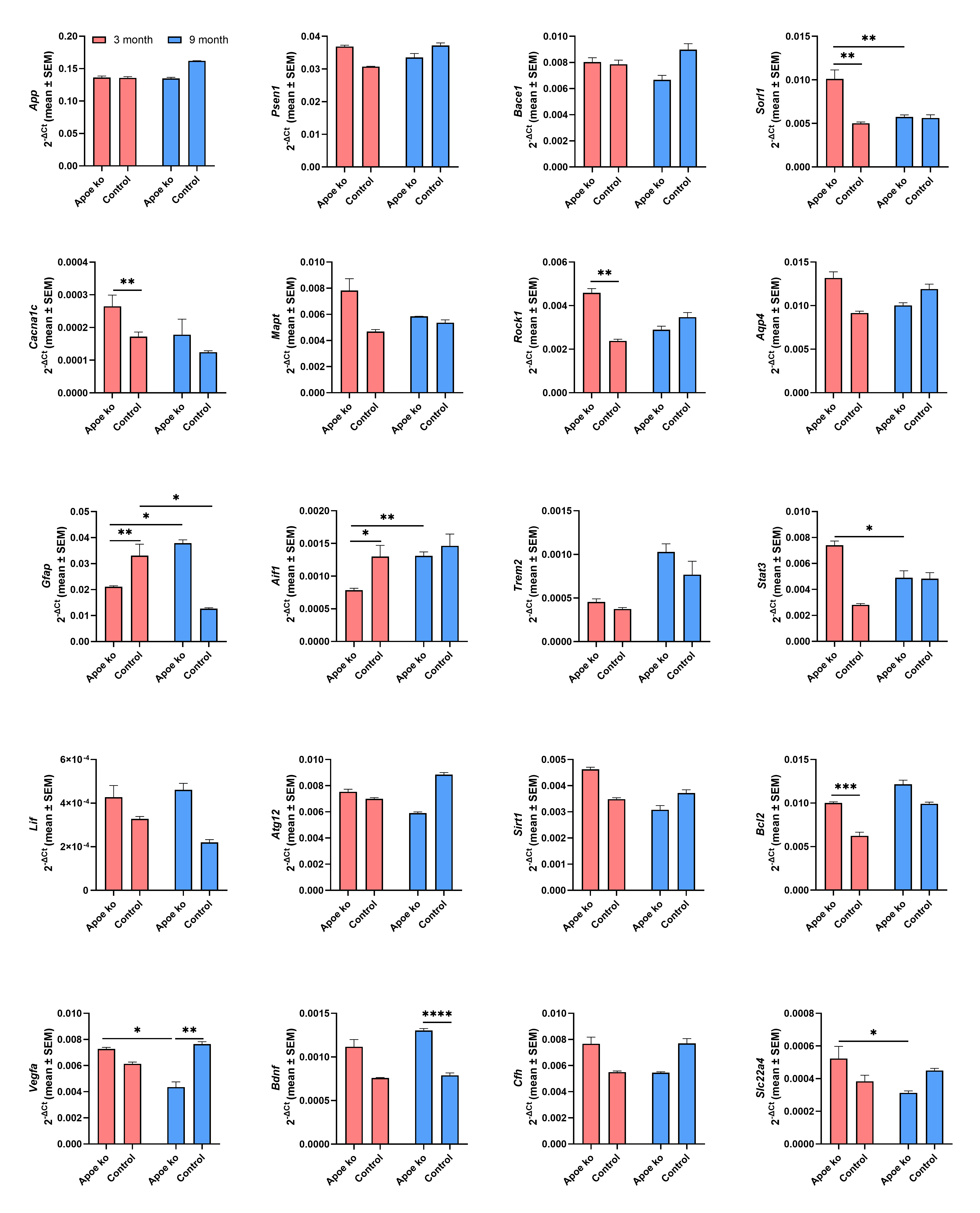

Supplement: SUPPLEMENTARY FIGURE S2 — Relative mRNA expression level in the eye tissue. Column graphs illustrate mean 2-∆Ct value across 3-month-old and 9-month-old Apoe-ko mice and controls. Pooled eye tissue sample (n = 4 per group) was used to determine the relative mRNA level. Differentially expressed target mRNA is defined based on a 1.5-fold and above intergroup difference and a sigficant P value at *P < 0.05, **P < 0.001, and ****P < 0.0001 (2-way ANOVA with Bonferroni corrected multiple comparisons test). Error bars indicate the standard error of the mean. [file Image_2.tif]

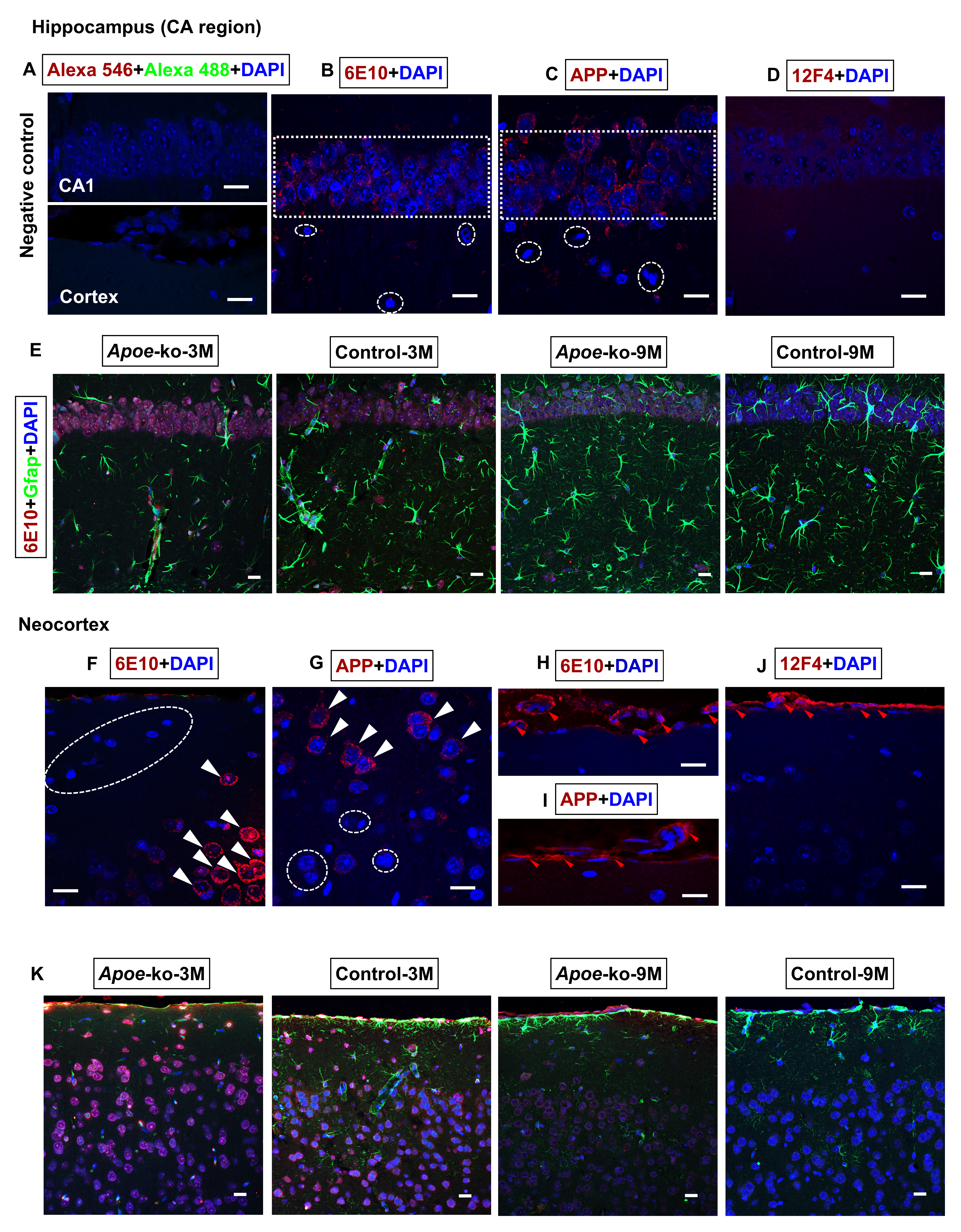

Supplement: SUPPLEMENTARY FIGURE S4 — Differences in APP/Aβ peptide and astroglia expression in the neocortex-hippocampus of Apoe-ko and control mice. (A) Negative control demonstrating the absence of APP/Aβ peptides and astroglia in the hippocampal and neocortical regions. Higher magnification images depict 6E10+ APP/Aβ peptides in the (B) CA region (white dashed box), (F) neocortex (white arrowheads), and (H) meninges (red arrowheads). White dashed circles indicate 6E10-negative cells. (C,G,I) APP presence was verified using a rabbit monoclonal APP antibody. (D,J) A mouse monoclonal antibody, 12F4, specific to the 1-42 amino acid residues of Aβ peptides, showed positive signals only in the meninges (red arrowheads). (E,K) Expression patterns of 6E10+ APP/Aβ peptides in the hippocampal CA1 region and neocortex of 3-month-old and 9-month-old Apoe-ko mice and controls. (Scale bar: 20 μm). [file Image_4.TIF]

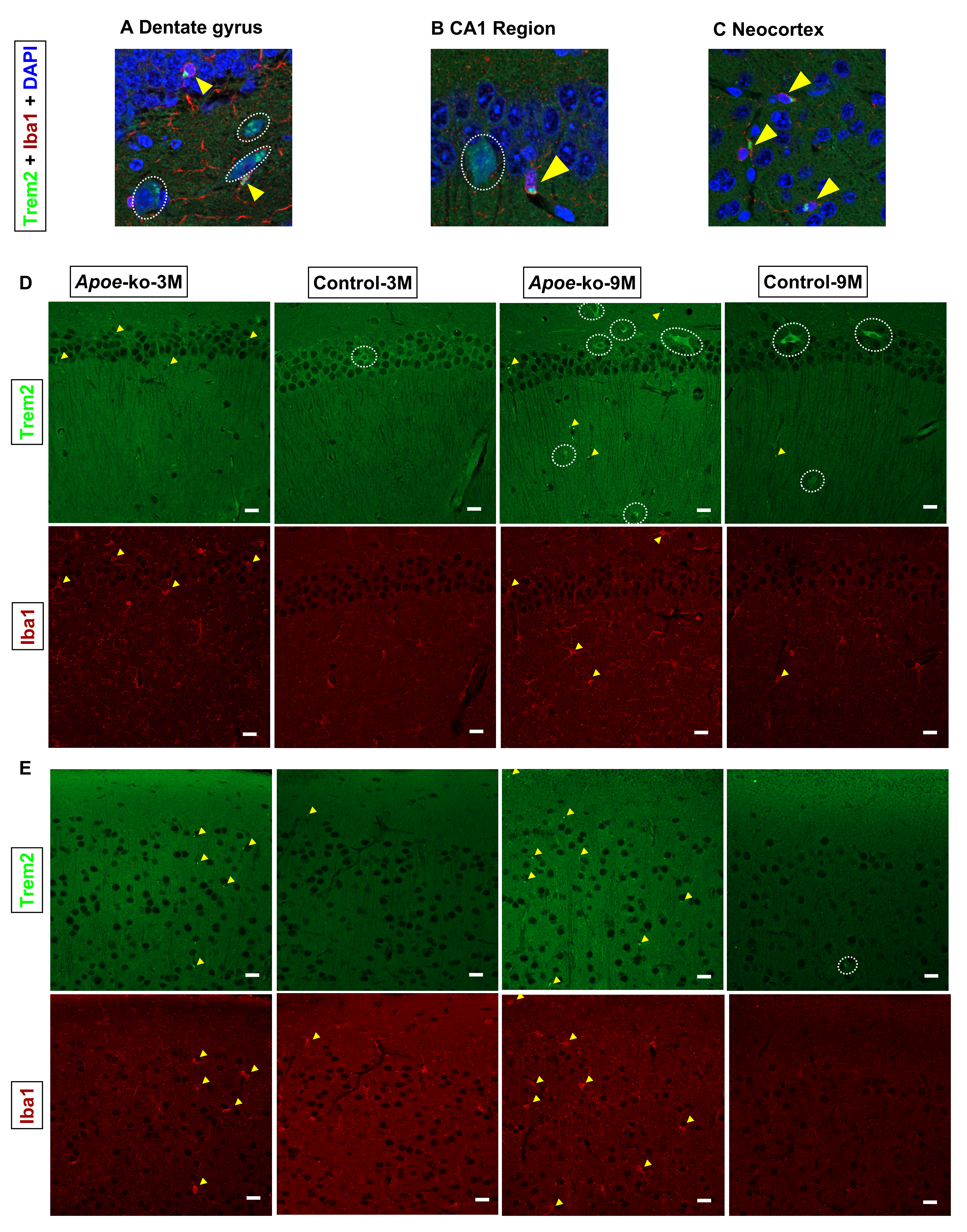

Supplement: SUPPLEMENTARY FIGURE S5 — Trem2 expression with and without microglial colocalization in the neocortex and hippocampus of Apoe-ko and control mice. (A–C) Colocalization of microglia expressing Trem2 receptors (yellow arrowheads) and cells expressing only Trem2 (white dashed circles) in the hippocampal dentate gyrus, CA1 region, and neocortex. (D,E) Expression patterns of Trem2 and Iba1+ microglia in Apoe-ko and control mice in the CA1 region of the hippocampus and the neocortex, respectively. White dashed circles (in green channel) indicate cells expressing only Trem2, while yellow arrowheads indicate Iba1+ microglia expressing Trem2 (in both green and red channels). (Scale bar: 20 μm). [file Image_5.TIF]

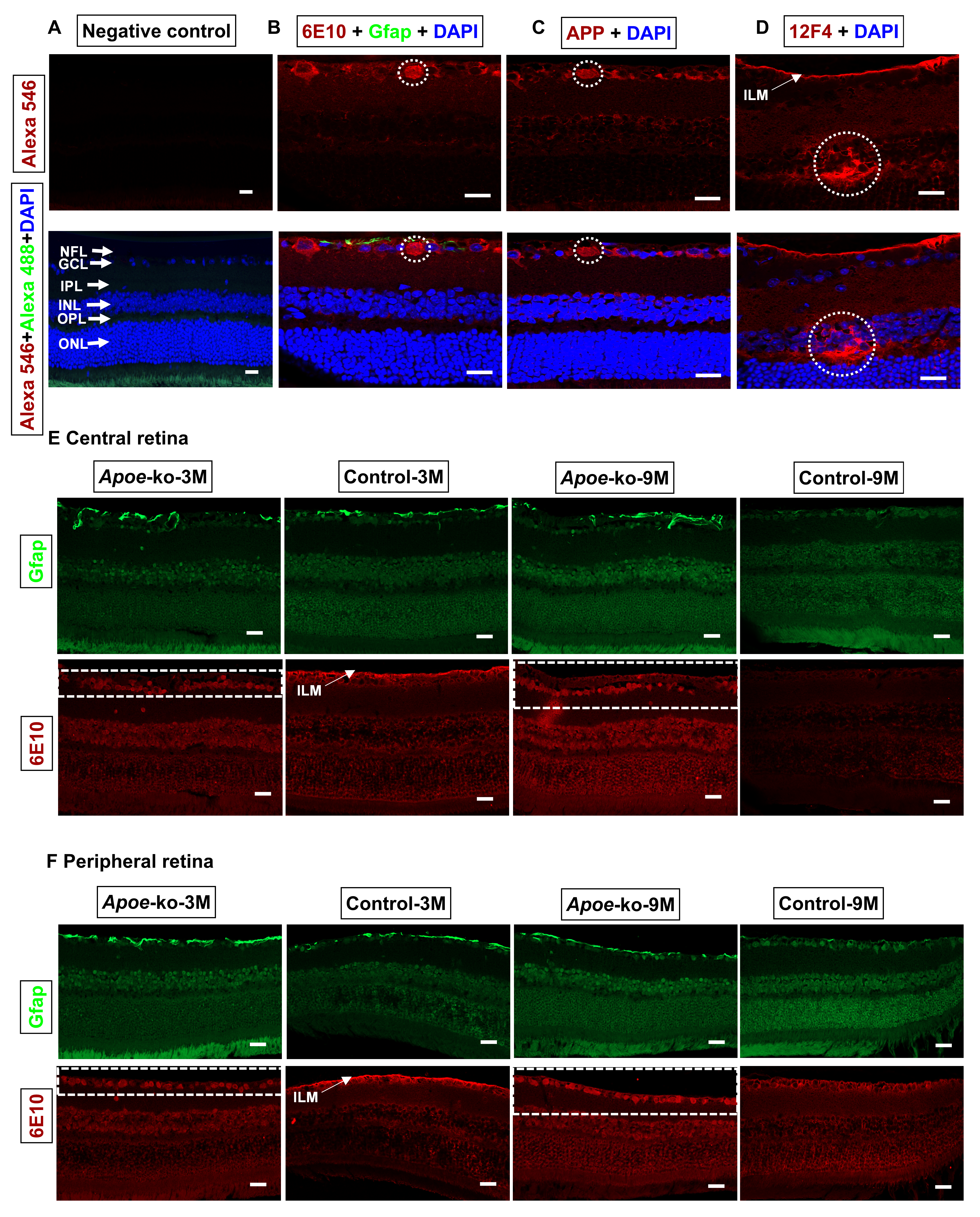

Supplement: SUPPLEMENTARY FIGURE S6 — Differences in APP/Aβ peptide and astroglial expression in the retina of Apoe-ko and control mice. (A) Negative control demonstrating the absence of APP/Aβ peptides and astroglia across retinal layers. (B) Higher magnification images showing 6E10+ APP/Aβ peptides predominantly within the GCL. (C) The presence of 6E10+ APP was confirmed using a rabbit monoclonal APP antibody in the GCL. A white dashed circle highlights diffuse deposition within the GCL in panels (B,C). (D) Additionally, 12F4 showed positive signals at the inner limiting membrane (ILM). The white dashed circle indicates a 12F4+ Aβ plaque-like deposition within the INL in panel D. (E,F) Expression patterns of 6E10+ APP/Aβ peptides within the central and peripheral retinas of 3-month-old and 9-month-old Apoe-ko mice and controls. White dashed boxes highlight the localization of 6E10+ APP/Aβ peptides within the GCL in both central and peripheral retinas. (Scale bar: 20 μm) (NFL, nerve fiber layer; GCL, ganglion cell layer; IPL, inner plexiform layer; INL, inner nuclear layer; OPL, outer plexiform layer; ONL, outer nuclear layer). [file Image_6.TIF]

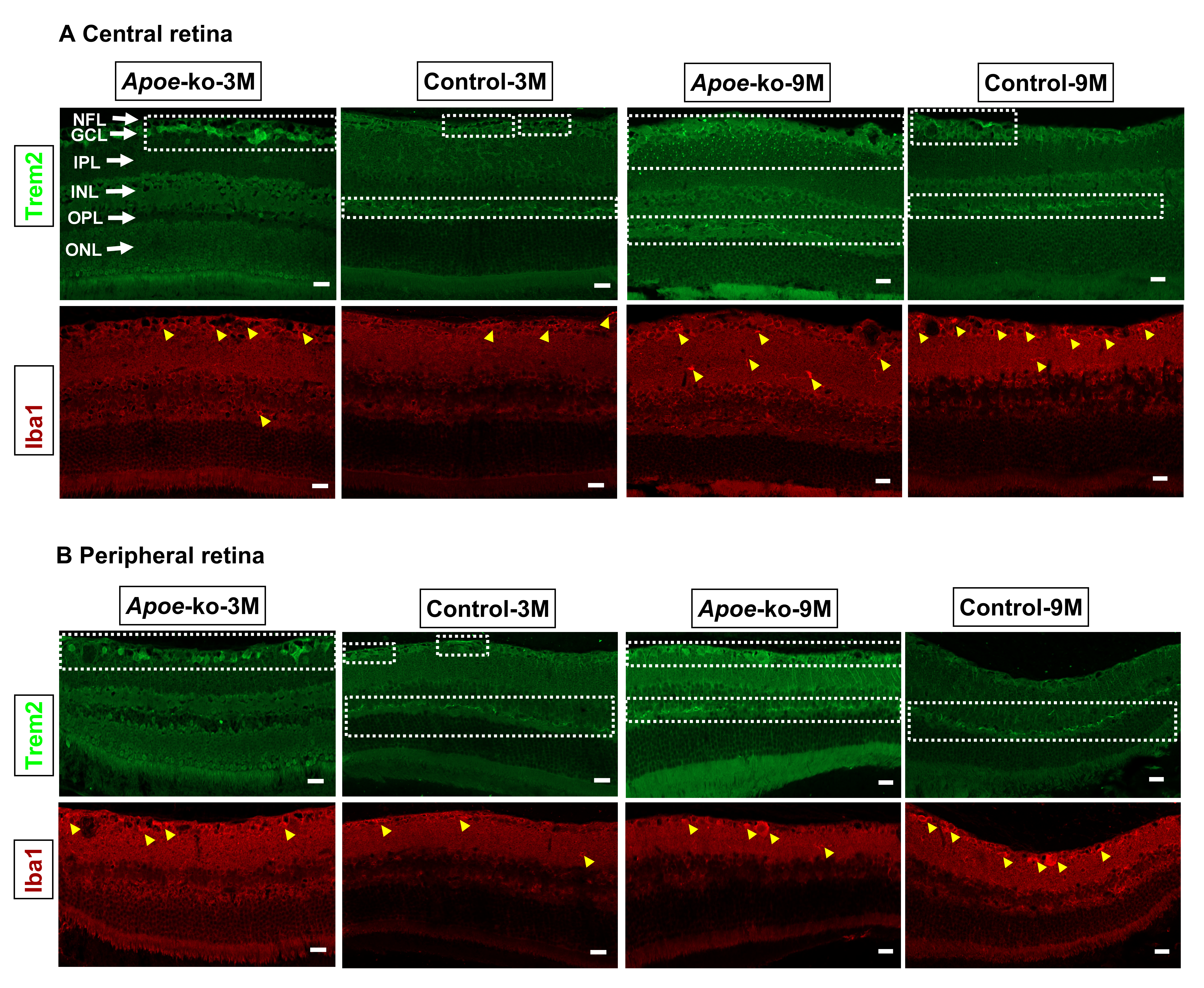

Supplement: SUPPLEMENTARY FIGURE S7 — Trem2 expression with and without microglia/macrophage colocalization across the retinal layers of Apoe-ko and control mice. (A,B) Expression patterns of Trem2 and Iba1+ microglia/macrophages in Apoe-ko and control mice are shown for the central and peripheral retinas, respectively. White dashed boxes indicate retinal layers expressing Trem2, while yellow arrowheads indicate Iba1+ microglia/macrophages within the retinal layers. Trem2 and Iba1+ microglia/macrophage expression, with or without colocalization, was predominantly identified in the NFL-GCL, followed by the OPL. (Scale bar: 20 μm) (NFL: nerve fiber layer; GCL: ganglion cell layer; IPL: inner plexiform layer; INL: inner nuclear layer; OPL: outer plexiform layer; ONL: outer nuclear layer). [file Image_7.TIF]
